# A Structural View on the Stereospecificity of Plant Borneol‐Type Dehydrogenases

**DOI:** 10.1002/cctc.202100110

**Published:** 2021-03-10

**Authors:** Andrea M. Chánique, Nicole Dimos, Ivana Drienovská, Elia Calderini, Mónica P. Pantín, Carl P. O. Helmer, Michael Hofer, Volker Sieber, Loreto P. Parra, Bernhard Loll, Robert Kourist

**Affiliations:** ^1^ Institute of Molecular Biotechnology Graz University of Technology Petersgasse 14 8010 Graz Austria; ^2^ Department of Chemical and Bioprocesses Engineering School of Engineering Pontificia Universidad Católica de Chile Vicuña Mackenna 4860 7810000 Santiago Chile; ^3^ Institute of Chemistry and Biochemistry Department of Biology Chemistry Pharmacy Laboratory of Structural Biochemistry Free University of Berlin Takustr. 6 14195 Berlin Germany; ^4^ Fraunhofer Institute for Interfacial Engineering and Biotechnology IGB Schulgasse 11a 94315 Straubing Germany; ^5^ Technical University of Munich Straubing Campus for Biotechnology and Sustainability Schulgasse 16 94315 Straubing Germany; ^6^ Institute for Biological and Medical Engineering Schools of Engineering Medicine and Biological Sciences Pontificia Universidad Católica de Chile Vicuña Mackenna 4860 7810000 Santiago Chile

**Keywords:** Enantioselectivity, *Salvia rosmarinus*, Terpenoids, Borneol, Short-chain dehydrogenase-reductase (SDR)

## Abstract

The development of sustainable processes for the valorization of byproducts and other waste streams remains an ongoing challenge in the field of catalysis. Racemic borneol, isoborneol and camphor are currently produced from α‐pinene, a side product from the production of cellulose. The pure enantiomers of these monoterpenoids have numerous applications in cosmetics and act as reagents for asymmetric synthesis, making an enzymatic route for their separation into optically pure enantiomers a desirable goal. Known short‐chain borneol‐type dehydrogenases (BDHs) from plants and bacteria lack the required specificity, stability or activity for industrial utilization. Prompted by reports on the presence of pure (−)‐borneol and (−)‐camphor in essential oils from rosemary, we set out to investigate dehydrogenases from the genus *Salvia* and discovered a dehydrogenase with high specificity (E>120) and high specific activity (>0.02 U mg^−1^) for borneol and isoborneol. Compared to other specific dehydrogenases, the one reported here shows remarkably higher stability, which was exploited to obtain the first three‐dimensional structure of an enantiospecific borneol‐type short‐chain dehydrogenase. This, together with docking studies, led to the identification of a hydrophobic pocket in the enzyme that plays a crucial role in the stereo discrimination of bornane‐type monoterpenoids. The kinetic resolution of borneol and isoborneol can be easily integrated into the existing synthetic route from α‐pinene to camphor thereby allowing the facile synthesis of optically pure monoterpenols from an abundant renewable source.

## Graphical abstract

**Highly Specific Borneol‐Type Dehydrogenase**: A borneol‐ type dehydrogenase with high specificity for borneol and isoborneol was characterized from the genome of *Salvia rosmarinus*. The crystallographic structure was determined, showing an hydrophobic pocket that plays a crucial role in the stereo discrimination of bornane‐type monoterpenoids.

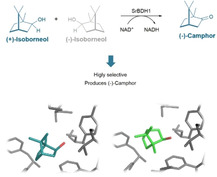

## Introduction

The enantiospecificity of many enzymes is a key feature that is currently exploited for numerous biotechnological processes. Often, increase or inversion of the specificity by enzyme engineering is necessary, for which an understanding of the molecular mechanisms of enantiospecificity becomes crucial. Several short‐chain dehydrogenases‐reductases (SDR) exert an intriguing two‐fold stereospecificity in the conversion of chiral monoterpenoids and could potentially be exploited for the synthesis of pure ingredients from essential oils.^[1]^ These enzymes catalyze the kinetic resolution of racemic alcohols and are also capable of forming a stereocenter by the asymmetric reduction of an enantiotopic or diastereotopic keto‐group.

**Scheme 1:**
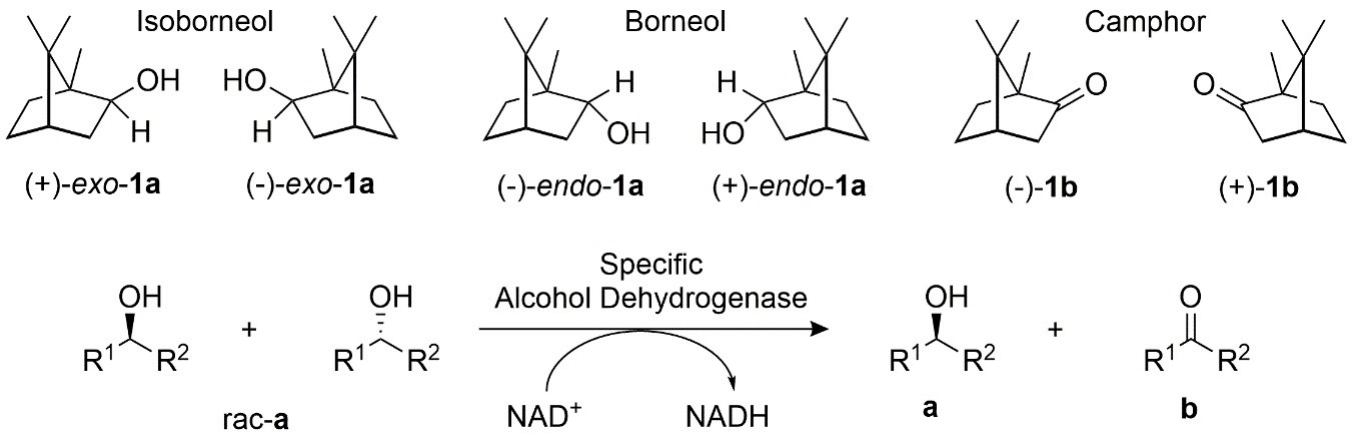
Chemical structures of the terpenoids *exo*‐**1 a**, *endo*‐**1 a** and **1 b** and generic oxidation catalyzed by a specific alcohol dehydrogenase.

Terpenes are a structurally and functionally diverse group of molecules. Their diversity is generated by the outstanding selectivity of the biosynthetic enzymes that participate in both the formation of the hydrocarbon skeleton and its oxyfunctionalization and further decoration.[^2^, ^3^] Among the vast diversity of bioactive terpenoids are the bornane‐type bicyclic monoterpenoids borneol (*endo*‐**1 a**) and isoborneol (*exo*‐**1 a**) and their corresponding ketone, camphor (**1 b**) (Scheme 1). They are found in essential oil extracts from different plants. Their pure isomers, and synthetic mixtures thereof, are widely used in Chinese medicine.^[4]^ Different studies have suggested their activity as anti‐inflammatory, neuroprotective and vasorelaxant agents, making them valuable ingredients for health‐related formulations.^[5]^ Derivatives of optically pure isoborneol such as (2*S*)‐(−)‐3‐*exo*‐(morpholino)isoborneol and (2S)‐(−)‐3‐exo‐(dimethylamino)isoborneol also find application as chiral ligands in asymmetric synthesis.[^6^, ^7^]

The biosynthesis of **1 b** proceeds from the cyclization and subsequent hydrolysis of geranyl diphosphate by borneol diphosphate synthase and borneol synthase.^[8]^ Enzymatic oxidation of **1 a** by an alcohol dehydrogenase then gives rise to **1 b**. This biosynthetic pathway does not necessarily require an enantiospecific borneol dehydrogenase as (+)‐*endo*‐**1 a** is produced from geranyl pyrophosphate already in optically pure form. In fact, the first recombinantly produced borneol dehydrogenases from the plants *Artemisia annua*[^9^, ^10^] and *Lavandula intermedia*,^[11]^ and the bacterium *Pseudomonas* sp. TCU‐HL1^[12]^ did not show any significant enantiospecificity, which seemed to confirm this notion.

Racemic **1 b** can easily be obtained by chemical synthesis from α‐ and β‐pinene with racemic **1 a** as intermediate.^[13]^ As α‐ and β‐pinene are side‐streams in the processing of pine trees, intermediates of this route represent inexpensive starting material for the production of optically pure isomers of bornane type monoterpenoids.^[13]^ This can be achieved by the kinetic resolution of *rac*‐**1 b** using a dehydrogenase that is enantiospecific towards **1 b** and diastereoselective for the formation of (+)‐*endo*‐**1 a**. Using iterative saturation mutagenesis, we recently created the first described variant of a bacterial short‐chain dehydrogenase capable of doing this.^[14]^ Alternatively, the oxidative kinetic resolution of *rac*‐*exo*‐**1 a** as intermediate of the existing chemical route is shorter and would provide a clean alternative to the isolation process from plants.^[15]^

In the 1980’s, Croteau and coworkers reported the presence of (+)‐specific borneol dehydrogenase activity in sage leaf homogenate (*Salvia officinalis L*.).^[16]^ Additionally, the essential oil from the related *Salvia rosmarinus* is reported to contain a high relative content of (−)‐*endo*‐**1 a** and (−)‐**1 b**.^[17]^ Based on the hypothesis that the synthesis of optically pure **1 a** and **1 b** isomers in *Salvia* species would require highly enantiospecific enzymes, we previously identified two dehydrogenases from *S. officinalis L*. that catalyze the specific oxidation of (+)‐*endo*‐**1 a** with outstanding enantiospecificity (E>200).^[1]^ The enantiopreference of these enzymes was at first sight unexpected, as the enzymes preferentially produced (+)‐**1 b** from racemic *endo*‐**1 a**, but (−)‐**1 b** from racemic *exo*‐**1 a**.^[1]^ Unfortunately, the low activity and stability of both enzymes represented an obstacle for synthetic application and structure elucidation. In order to find an enzyme with higher stability and to understand whether enantiospecificity is a frequent feature or an exception in this group of dehydrogenases, we continued studying other enzymes from the *Salvia* genus, specifically, *S. rosmarinus* and *S. officinalis*. We also investigated the capability of these plant dehydrogenases to perform the reverse reaction. Herein, we report the specificity, substrate scope and activity of a set of plant borneol‐type dehydrogenases, with special emphasis on the highly stable and active borneol dehydrogenase SrBDH1 from *S. rosmarinus*. We also determined the structure of SrBDH1 in complex with NAD^+^ at 2.8 Å resolution. This represents the first structure of a selective borneol‐type SDR; from which a deeper insight into the molecular basis of the enantiospecificity of the enzyme was obtained.

## Results and discussion

### Sequence analysis

**Figure 1:**
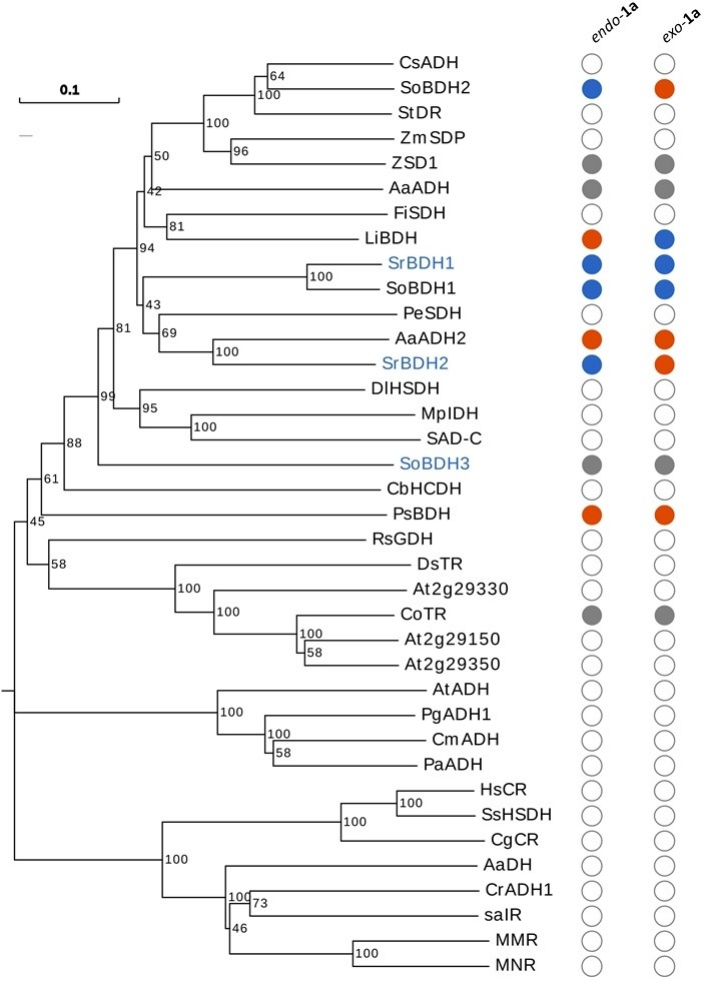
Phylogram showing evolutionary relationships of different short‐chain dehydrogenases. The tree was constructed using Maximum Likelihood method with Mega X software. The three borneol‐type dehydrogenases characterized in this paper are highlighted in blue. Specificity for *endo*‐**1 a** and *exo*‐**1 a** is indicated in circles, with high specificity in blue (E>100) and low specificity in orange. Enzymes described to convert **1 a** with no indicated specificity are shown with grey circles and enzymes not described to convert it are shown with white. The bootstrap values are shown next to each branch. For the sequences of short‐chain dehydrogenases and their accession numbers, please refer to the Supplementary Information.

Two putative members of the SDR class from the genome *S. rosmarinus* and one from *S. officinalis L*.^[18]^ were identified using the BLASTP server. The percentage of identity with the unselective AaADH2 (*Artemisia annua*)^[10]^ ranged between 43 % and 53 %, making them likely candidates for borneol‐converting dehydrogenases. For *S. officinalis L*., the two first hits had already been shown to convert (+)‐*endo*‐**1 a** in an enantiospecific fashion (SoBDH1 and SoBDH2).^[1]^ The three putative dehydrogenases have the typical TGxxx[AG]xG cofactor binding motif and the YxxxK active site motif that is characteristic to the classic SDR family (Figure S1).^[19]^ According to the classification suggested by Kallberg *et al*. (2010)^[20]^ all of the alcohol dehydrogenases investigated in this paper (except the bacterial borneol dehydrogenase from *Pseudomonas* sp. TCU‐HL1 (PsBDH)) belong to the SDR110 C subgroup of the SDR superfamily (hereafter, borneol‐type dehydrogenases). Other members are the sex determination protein tasselseed‐2 from *Zea mays* (ZmSDP) and secoisolariciresinol dehydrogenase from *Podophyllum peltatum* (PeSDH), which show a wide diversity in terms of function and substrate acceptance within the subgroup (Figure 1). Other subgroups displayed in the phylogenetic tree are tropinone reductase‐like SDRs (SDR65 C), menthol dehydrogenase‐like SDRs (SDR114 C) and carbonyl reductases (SDR21 C).

### Recombinant production and substrate scope

**Figure 2:**
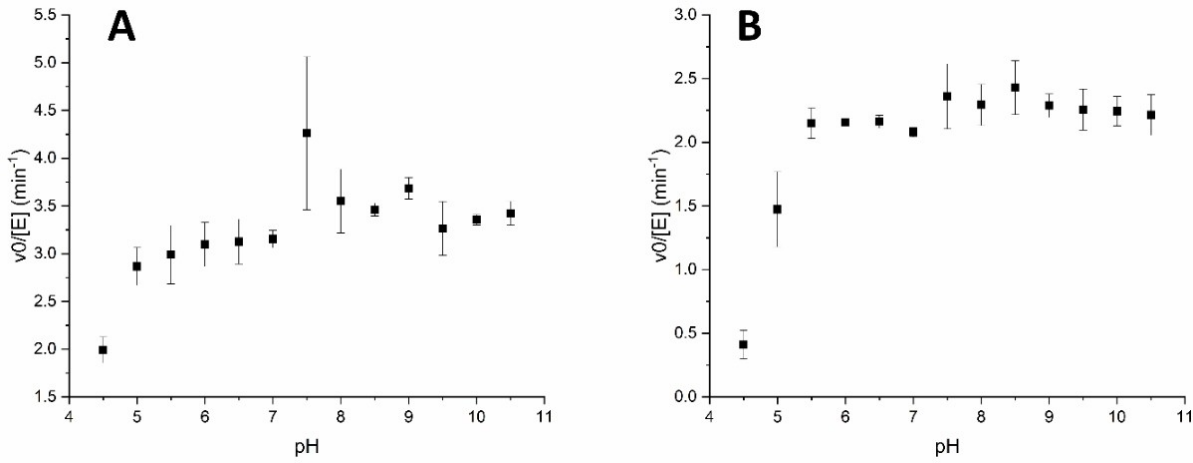
Rates for conversion of (+)‐*endo*‐**1 a** for SrBDH1 after incubation at different pH values. The enzyme was incubated at room temperature for 30 min (**A**) and 24 h (**B**) at the indicated pH and then the initial rate of oxidation of (+)‐*endo*‐**1 a** was determined at pH 9 by following NADH formation at 340 nm.

The recombinant production in *E. coli* and subsequent purification of the three enzymes yielded ∼50 mg L^−1^ for SrBDH1, and values in the same range for SrBDH2 and SoBDH3 (Figure S2). Size exclusion chromatography classifies the enzymes as tetramers (Figure S3). SrBDH1 had a specific activity of 0.030 U mg^−1^ towards *rac*‐*endo*‐**1 a**, the highest value among borneol‐type dehydrogenases from plants. pH stability analysis showed over 50 % retention of activity for pH values between 5 and 10.5 after 30 min of incubation at the selected pH (Figure 2A). After 24 h of incubation, the activity showed little variation for pH between 5.5 and 10.5 (Figure 2B). This relatively high stability at a broad pH range is particularly interesting for reduction reactions, which are favored at an acidic pH. It is also observed from Figure 2 that after 24 h of incubation at neutral pH values, the enzyme still retained 50 % of its initial activity. This is a very promising starting point for further optimization by protein and reaction engineering. The higher stability of SrBDH1 in comparison to previously investigated enzymes from *S. officinalis L*., also allowed us to successfully elucidate its crystal structure.

**Figure 3:**
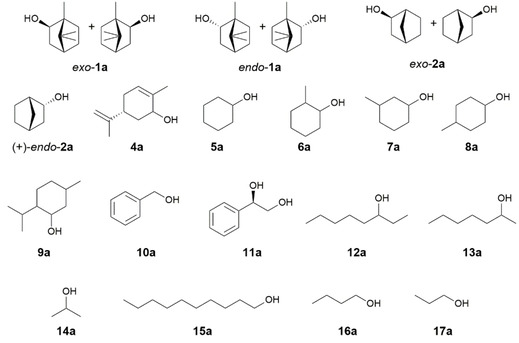
Primary and secondary alcohols tested in the substrate scope study of BDHs.

Determination of the specific activities of the BDHs in the oxidation of a set of primary and secondary alcohols showed that the enzymes do not exclusively oxidize bicyclic monoterpenols; they also accept monocyclic and linear substrates (Figure 3, Table 1). The dehydrogenases clearly favor secondary alcohols over primary alcohols. All enzymes had the highest specific activity either for *endo*‐**1 a** (SrBDH1 and PsBDH), (−)‐carveol (**4 a**) (SrBDH2, SoBDH3 and AaADH2) or 3‐methyl cyclohexanol (**7 a**) (SoBDH2).

**Table 1:** Specific activities measured for different borneol‐type dehydrogenases.

	Specific activity [mU/mg] ^[a]^					
Substrate	SrBDH1^[b]^	SrBDH2^[b]^	SoBDH3^[b]^	AaADH2^[b]^	SoBDH2^[b]^	PsBDH^[b]^
exo‐**1 a**	24	5.1	0.3	53	13	115
endo‐**1 a**	**30**	10.0	n.c.^[c]^	22	18	**122**
exo‐**2 a**	4.8	1.5	n.c.	29	n.c.	69
(+)‐endo‐**2 a**	3.9	5.9	n.c.	23	n.d.	28
endo‐**3 a**	4.6	1.0	n.c.	14	7.1	30
**4 a**	2.9	**16**	**0.46**	**88**	12	5.0
**5 a**	3.3	n.c.	n.c.	29	6.7	28
**6 a**	10	2.5	n.c.	17	18	23
**7 a**	4.4	n.c.	0.23	21	**39**	18
**8 a**	8.3	n.c.	0.21	15	3.8	11
**9 a**	n.c.	n.c.	n.c.	n.c.	n.c.	n.c.
**10 a**	n.c.	n.c.	n.c.	5.9	n.c.	n.c.
**11 a**	n.c.	n.c.	n.c.	n.c.	n.c.	n.c.
**12 a**	13	4.4	n.c.	8.7	2.3	43
**13 a**	9.8	2.0	n.c.	63	6.3	6.2
**14 a**	n.c.	n.c.	n.c.	10.0	n.c.	n.c.
**15 a**	n.c.	n.c.	n.c.	n.c.	3.4	n.c.
**16 a**	n.c.	n.c.	n.c.	4.8	4.7	n.c.
**17 a**	3.3	n.c.	0.2	n.c.	6.5	3.2

[a] The highest activity for each enzyme is highlighted in bold letters. For the experimental error from technical triplicates, please refer to **Table S1**, [b] AaADH2: alcohol dehydrogenase from *A. annua* ^[10]^; SoBDH2: borneol dehydrogenase from *S. officinalis L*.^[1]^; SrBDH1/2: borneol‐ like dehydrogenases from *S. rosmarinus*; SoBDH3: borneol‐like dehydrogenase from *S. officinalis L*.; PsBDH: borneol dehydrogenase from *Pseudomonas* sp. TCU‐HL1.^[12],^ [c] n.c.=no conversion detected. Reactions with substrates displaying specific activities less than twice the blank are not shown.

Interestingly, we noted that none of the enzymes oxidized menthol (**9 a**), despite its structural similarity to **4 a**. In fact, menthol dehydrogenases appear as a separated clade in the phylogram (Figure 1, MMR and MNR). A BLASTP search using menthol and neomenthol dehydrogenases from *Mentha x piperita* as queries suggests that this subfamily of SDRs is also present in *S. officinalis* and *S. rosmarinus* (best hits with 67 % identity for MNR and 68 % identity for MMR), leading us to think that *Salvia* plants have independent biocatalysts for the synthesis of menthone‐like compounds and camphor‐like compounds. We also noted differences in the specific activities obtained for the unnatural substrate cyclohexanol (**5 a**) and the isomers 2‐methylcyclohexanol (**6 a**), 3‐methylcyclohexanol (**7 a**) and 4‐methylcyclohexanol (**8 a**). For SrBDH1, SrBDH2 and SoBDH2 we observed a higher specific activity for **6 a** in comparison to **5 a**. This situation is similar to what we observed with *endo*‐**1 a** vs *endo*‐**2 a**, where the presence of methyl groups seems to improve the fit in the active site, making the reaction faster. Conversely, the unselective enzymes AaADH2 and PsBDH have a higher specific activity for the alcohol without the methyl group, **5 a**. The specific activities observed for SrBDH1 with *endo*‐**1 a** and *exo*‐**1 a** are the best among the selective enzymes studied and fall within the same range of the unselective borneol dehydrogenase from *A. annua* AaADH2, making SrBDH1 interesting for biocatalytic applications. Cofactor usage was also studied spectrophotometrically. Results showed that AaADH2 was the only enzyme also capable of using NADP^+^, albeit less efficiently than NAD^+^. The other tested enzymes did not show measurable activity for NADP^+^ (Figure S4).

### Enantiospecificity and selectivity towards bicyclic alcohols and ketones

**Scheme 2:**
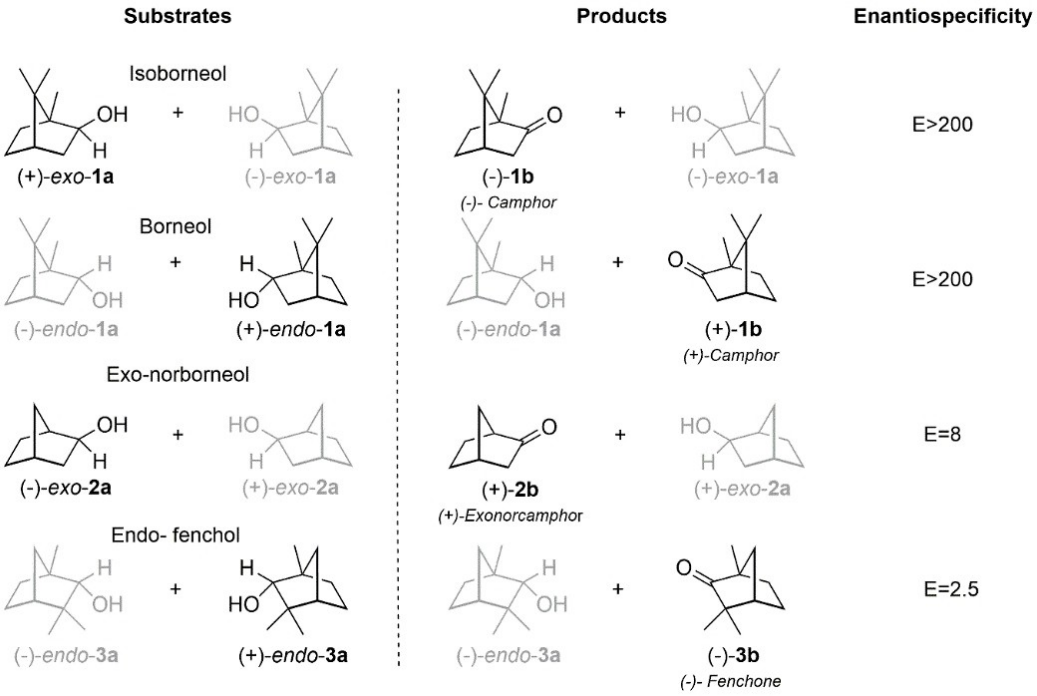
Preferred substrates (highlighted in black) and enantiospecificity of SrBDH1 in the kinetic resolution of bicyclic secondary alcohols.

To obtain a more systematic overview on the enantiospecificity of the dehydrogenases, we investigated the kinetic resolution of the three bornane type monoterpenols *endo*‐**1 a**, *exo*‐**1 a** and fenchol (*endo*‐**3 a**) and the structurally related *exo*‐norborneol (*exo*‐**2 a**) (Table 2, Scheme 2). SoBDH1 and SrBDH1 showed outstanding enantiospecificity (E>200) towards both *endo*‐**1 a** and *exo*‐**1 a**, while SoBDH2 and SrBDH2 showed specificity for *endo*‐**1 a**, but not for *exo*‐**1 a**. The differences in the specificity of this set of enzymes towards *exo*‐**1 a** and the smaller *exo*‐**2 a** were striking. In particular, SoBDH1 and SrBDH1 were hardly specific towards *exo*‐**2 a**, in contrast with *exo*‐**1 a**, which might be an indication that a precise fit of the substrate is important for the specificity of both enzymes. It should be noted, however, that the activity of SrBDH1 towards *exo*‐**2 a** is substantially lower than towards *exo*‐**1 a**, making comparisons of the specificity difficult. SrBDH1 shows a similar drop in activity and specificity in the conversion of the structural isomer *endo*‐**3 a** (Table 2, Scheme 2). The differences in activity and selectivity of SrBDH1 towards these substrates indicates that the position of the methyl substituents is crucial for the substrate recognition of this enzyme. Therefore, the high specificity of SrBDH1 and SoBDH1 towards both *endo*‐**1 a** and *exo*‐**1 a** is somehow counterintuitive and led us to think that these two enzymes share key active pocket configurations that other BDHs lack. None of the plant dehydrogenases have any noteworthy specificity in the resolution of *endo*‐**3 a**. While the investigated enzymes share the enantiopreference for the same isomer of *endo*‐**1 a**, *exo*‐**1 a** and *exo*‐**2 a**, we noted that the preference for the (+) and (−) isomers of *endo‐* **3 a** differed.

**Table 2:** Kinetic resolution of racemic secondary alcohols of the bornane and norbornane types catalyzed by alcohol dehydrogenases at a substrate concentration of 1 mM.

			AaADH2^[a]^		SoBDH1 ^[a]^		SoBDH2^[a]^		SoBDH3^[b]^		SrBDH1^[b]^		SrBDH2^[b]^			PsBDH ^[a]^
																
*exo*‐**1 a**	Time (h)		0.5		24		4		48		2		48			0.5
	Specificity		(+)		(+)		(+)		(+)		(+)		(+)			(+)
	%ee_p_^[c]^		4 %		99 %		13 %		>99 %		>99 %		31 %			4 %
	%ee_s_^[c]^		99 %		30 %		99 %		5 %		52 %		32 %			27 %
	%c		96 %		28 %		88 %		5 %		34 %		51 %			87 %
	E^[f]^		n.d.		>200		4.6		n.d.		>200		3			1.3
																
*endo*‐**1 a**	Time (h)		0.5		24		4		n.c.^[d]^		2		48			0.5
	Specificity		(+)		(+)		(+)		n.c.		(+)		(+)			(+)
	%ee_p_^[c]^		46 %		>99 %		>99 %		n.c.		>99 %		>99 %			17 %
	%ee_s_^[c]^		83 %		18 %		49 %		n.c.		77 %		23 %			50 %
	%c		64 %		22 %		33 %		n.c.		44 %		19 %			75 %
	E^[f]^		6.6		>200		>200		n.d. ^[e]^		>200		>200			2.1
																
*exo*‐**2 a**	Time (h)		0.25		48		48		n.c.		48		48			0.25
	Specificity		(−)		(−)		(−)		n.c.		(−)		(−)			(−)
	%ee_p_^[c]^		65 %		10 %		80 %		n.c.		75 %		49 %			9 %
	%ee_s_^[c]^		53 %		3 %		15 %		n.c.		10 %		1 %			2 %
	%c		45 %		22 %		16 %		n.c.		12 %		2 %			18 %
	E^[f]^		7.9		1.2		10.3		n.d.		7.7		n.d			1.2
																
*endo*‐**3 a**	Time (h)		0.25		n.c.		48		48		48		48			48
	Specificity		(+)		n.c.		(−)		n.d.		(+)		(−)			(+)
	%ee_p_^[c,g]^		‐		n.c.		‐		‐		‐		‐			‐
	%ee_s_^[c]^		13 %		n.c.		84 %		<1 %		26 %		6 %			>99 %
	%c		67 %		n.c.		58 %		22 %		45 %		10 %			59 %
	E^[f]^		1.3		n.d.		10		1		2.5		3.7			27

[a] Data from literature for *endo*‐**1 a** and *exo*‐**1 a**.^[1]^; AaADH2: alcohol dehydrogenase from *A. annua*.^[10]^; SoBDH1/2: borneol‐type dehydrogenases from *S. officinalis* L.^[1]^; PsBDH: borneol dehydrogenase from *Pseudomonas* sp. TCU‐HL1,^[12]^ [b] SoBDH3: borneol‐ type dehydrogenases from *S. officinalis*; SrBDH1/2: borneol‐ type dehydrogenases from *S. rosmarinus*, [c] %ee_s_: Enantiomeric excess of substrates. %ee_p_: Enantiomeric excess of the products. All enantiomeric excess were determined by chiral gas chromatography. [d] n.c.: no conversion, [e] n.d.: not determined, [f] Calculated according to Straathop and Jogejan (1997).^[53]^ [g] %ee_p_ not calculated as we did not obtain baseline separation for the fenchone enantiomers in GC analysis.

PsBDH displays a different behavior in comparison to plant BDHs, as the bacterial enzyme showed very low specificity towards *endo*‐**1 a**, *exo*‐**1 a** and *exo*‐**2 a**, but it is the most specific of the studied enzymes for the oxidation of *endo*‐**3 a**, with >99 % ee_s_ and E=27. SoBDH3 was not active towards *endo*‐**1 a** and *exo*‐**2 a** and showed little conversion for *exo*‐**1 a**, leading us to assume that this enzyme has a natural substrate with significant structural differences from *endo*‐**1 a**, which is reflected in the divergent sequence compared to other BDHs from plants (Figure 1).

**Scheme 3:**
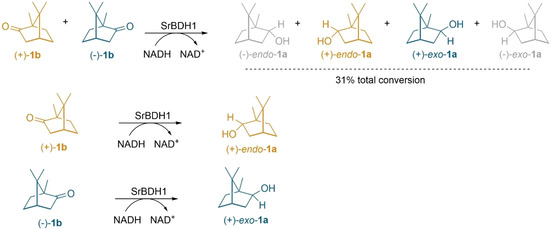
Reduction of racemic and pure enantiomers of **1 b** by SrBDH1 at pH 5.5 with phosphite dehydrogenase as cofactor‐regeneration system. Reaction with 5 mM of substrate, 20 μM of NADH, 10 mM of phosphite and 12 μM of phosphite dehydrogenase.

Based on reports describing a short‐chain dehydrogenase from the tropinone reductase subfamily capable of catalyzing the stereoselective reduction of pure **1 b** isomers,^[21]^ and an engineered *Pseudomonas* dehydrogenase with high enantiospecificity towards (+)‐**1 b**,^[14]^ we decided to investigate if borneol‐type dehydrogenases were also capable of catalyzing the reduction. The reduction reaction is favored at acidic pH values, however, we used pH 5.5 as the stability of SrBDH1 was notably reduced at lower pH values (Figure 2). In fact, all of the enzymes showed some degree of precipitation at pH 5.0. Among borneol‐type dehydrogenases, AaADH2 and SrBDH1 were the only ones that catalyzed the reduction of **1 b** (Table 3). SrBDH1 exclusively produced (+)‐*endo*‐**1 a** from pure (+)‐**1 b** and (+)‐*exo*‐**1 a** from pure (−)‐**1 b** (de_p_>99 % for both) (Table S2). As the enzyme converts both enantiomers of **1 b**, reduction of rac‐**1 b** produces a mixture of (+)‐*endo*‐**1 a** and (+)‐*exo*‐**1 a** in the ratio 91 : 9. AaADH2 showed lower diastereoselectivity and produced (+)‐*endo*‐**1 a** with 89 % de_p_ and (+)‐*exo*‐**1 b** with 94 % de_p_, respectively (Scheme 3, Figure S5). Intriguingly, all of the enzymes, except SrBDH1, showed a higher conversion for **2 b** in comparison to **1 b**, while for the oxidations, we obtained better conversion for *endo*‐**1 a** and *exo*‐**1 a** compared to *exo*‐**2 a**. Also from the DR in the reductions, we observe *endo* predominance in all cases, leading us to think that *endo*‐**2 a** would be better oxidized than *exo*‐**2 a** for the studied enzymes. In the case of **3 b**, the only enzyme catalyzing the reduction was AaADH2. This last result also contrasts with the capacity of catalyzing the oxidation of *endo*‐**3 a** observed in all the studied enzymes, and could be indicating, for instance, an inhibiting effect caused by *exo*‐**3 a**, which we did not analyze for oxidation reactions.

**Table 3:** Reduction of racemic bicyclic ketones catalyzed by alcohol dehydrogenases at a substrate concentration of 5 mM.

			AaADH2^[a]^		SoBDH1^[a]^		SoBDH2^[a]^		SoBDH3^[a]^		SrBDH1^[a]^		SrBDH2^[a]^			PsBDH^[a]^
																
*rac*‐**1 b**	Time (h)		48		n.c.^[e]^		n.c.		n.c.		48		n.c.			48
	Specificity		(+)		n.c.		n.c.		n.c.		(+)		n.c.			(+)
	%c		51 %		n.c.		n.c.		n.c.		31 %		n.c.			22 %
	%ee_s_^[b]^		10 %		n.c.		n.c.		n.c.		36 %		n.c.			11 %
	%ee_p_^[b]^		10 %		n.c.		n.c.		n.c.		80 %		n.c.			37 %
	E ^[c]^		1.3		n.c.		n.c.		n.c.		12.4		n.c.			2.4
	DR^[d]^		44 : 3 : 51 : 2		n.c.		n.c.		n.c.		91:0 : 9:0		n.c.			65 : 24 : 5 : 5
																
*rac*‐**2 b**	Time (h)		4		48		48		n.c.		48		48			48
	Specificity		(+)		(−)		(−)		n.c.		(−)		(−)			(−)
	%c		94 %		2 %		7 %		n.c.		30 %		2 %			88 %
	%ee_s_^[b]^		60 %		<1 %		69 %		n.c.		33 %		1 %			38 %
	%ee_p_^[b]^		4 %		19 %		85 %		n.c.		76 %		51 %			5 %
	E ^[c]^		1.6		n.d.^[f]^		5.8		n.c.		10		n.d.			1.4
	DR^[d]^		13 : 41 : 7 : 39		36 : 41 : 18 : 4		10 : 85:0 : 6		n.c.		6 : 87:0 : 6		14 : 68 : 7 : 10			22 : 37 : 16 : 26
																
*rac*‐**3 b**	Time (h)		48		n.c.		n.c.		n.c.		n.c.		n.c.			n.c.
	Specificity		(+)		n.c.		n.c.		n.c.		n.c.		n.c.			n.c.
	%c		49 %		n.c.		n.c.		n.c.		n.c.		n.c.			n.c.
	%ee_s_^[b]^		9 %		n.c.		n.c.		n.c.		n.c.		n.c.			n.c.
	%ee_p_^[b]^		9 %		n.c.		n.c.		n.c.		n.c.		n.c.			n.c.
	E ^[c]^		1.3		n.c.		n.c.		n.c.		n.c.		n.c.			n.c.
	DR^[d]^		40 : 54 : 5:0		n.c.		n.c.		n.c.		n.c.		n.c.			n.c.

[a] AaADH2: ADH from *A. annua*.^[10]^; SoBDH2: borneol dehydrogenase from *S. officinalis* L.^[1]^; SrBDH1/2: borneol‐ like dehydrogenases from *S. rosmarinus*; SoBDH3: borneol‐like dehydrogenase from *S. officinalis* L. PsBDH: borneol dehydrogenase from *Pseudomonas* sp. TCU‐HL1.^[12]^ [b] %ee_s_: percentage enantiomeric excess of substrates. %ee_p_: percentage enantiomeric excess of the products. All enantiomeric excess were determined by chiral gas chromatography. The enantiomeric excess of the product was calculated based on the sums of the products resulting from (+)‐**1 b** and (−)‐**1 b**, (+)‐**2 b** and (−)‐**2 b** or (+)‐**3 b** and (−)‐**3 b**, respectively. For instance, the ee_p_ for the conversion of **1** is defined as ee_p_=(([(+)‐*exo*‐**1 a**]+[(−)‐*endo*‐**1 a**])‐([(−)‐*exo*‐**1 a**]+[(+)‐*endo*‐**1 a**]))/(([(+)‐*exo*‐**1 a**]+[(−)‐*endo*‐**1 a**])+([(−)‐*exo*‐**1 a**]+[(+)‐*endo*‐**1 a**])), [c] calculated according to Straathop and Jogejan (1997),^[53]^ [d] DR: diastereomer ratio: (+)‐*endo*‐**a** : (−)‐*endo*‐**a** : (+)‐*exo*‐**a** : (−)‐*exo*‐**a**. [e] n.c.: no conversion. [f] n.d. : not determined;

### Kinetic parameters of SrBDH1

The high activity of SrBDH1 stood out amongst this family of enzymes. While the poor solubility of (+)‐*endo*‐**1 a** limited the determination of initial rates to concentrations up to 6 mM (three‐fold of the K_M_‐value), the apparent *k*_obs_ obtained corresponds to 0.20 s^−1^ (Figure S6). This value is higher by at least two orders of magnitude compared with those of related dehydrogenases from *S. officinalis L*. (0.005 s^−1^),^[1]^ *A. annua* (0.006 s^−1^),^[9]^ *L. intermedia* (0.0004 s^−1^)^[11]^ and one order of magnitude compared with tropinone reductase from *Cochlearia officinalis* CoTR (0.09 s^−1^ towards (−)‐*endo*‐***1 a***).^[21]^ The value is comparable to the non‐specific bacterial PsBDH from *Pseudomonas* sp. TCU‐HL1 (0.75 s^−1^).^[12]^ The *K*_M_‐value of 2.02±0.18 mM is surprisingly high compared to those of the other plant dehydrogenases (typically 50 μM^[1]^). At a non‐saturating concentration of (+)‐*endo*‐**1 a**, the *K*_M_‐value estimated for NAD^+^ was 100±26 μM, which is comparable to the *K*_M_ for NAD^+^ determined for SoBDH2.^[1]^

### Structure elucidation and comparison with PsBDH of Pseudomonas sp. TCU‐HL1

**Figure 4:**
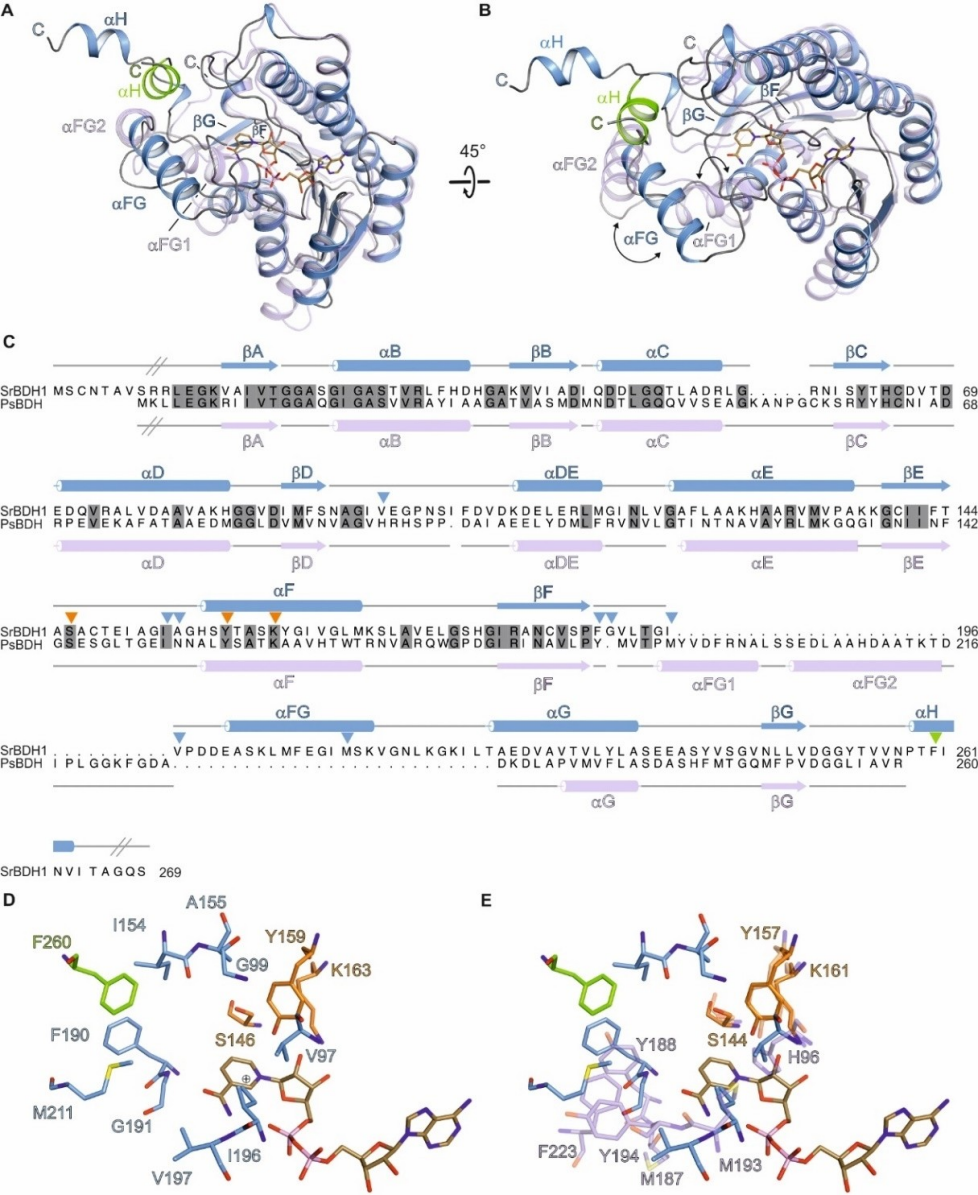
**(A)** In cartoon representation, a superposition of one monomer of SrBDH1 and PsBDH. Identical view as in Figure S7. SrBDH1 is shown in blue and PsBDH in violet. The C‐terminal α‐helix (αH) of a symmetry‐related molecule, that shields the active site, is shown in green cartoon representation. The NAD^+^ bound to SrBDH1 is shown as brown sticks. **(B)** View of panel **(A)** rotated by 45°. **(C)** Structure‐based sequence alignment of SrBDH1 and PsBDH PDB‐ID: 6M5N. The secondary structure elements are shown above the alignment for SrBDH1 and below for PsBDH with α‐helices depicted as cylinders and β‐strands as arrows. Inclined lines indicated sections of SrBDH1 and PsBDH, that are not included in modelled the crystal structures. The catalytic motif is indicated by orange triangles. Amino acids lining the putative active site of SrBDH1 are indicated by blue triangles and as green triangle if derived for another SrBDH1 monomer within the tetramer. **(D)** Stick representation of residues lining the active site of SrBDH1 with bound NAD^+^. The active is site is defined mainly by hydrophobic amino acid residues from two symmetry‐related protein monomers colored in blue and green, respectively. **(E)** Stick representation of residues lining the active site of chain C with the bound NAD^+^ and PsBDH in violet. The active is site is defined mainly by hydrophobic amino acid residues from two different protein monomers. Color‐coding according to panel A.

SrBDH1 crystals were obtained under two different crystallization conditions. The first one with high concentrations of NaCl and the second one using the polymer pentaerythritol propoxylate (PO/OH). The obtained crystals belong to two different space groups (Table S3) with different crystal packing. The overall architecture of SrBDH1 *in crystallo* is tetrameric (Figure S7), in agreement with SEC/MALS measurements confirming a tetrameric state in solution (Figure S8). The tetramers as obtained from both crystallization conditions are practically undistinguishable with a root mean square deviation (RMSD) of 0.435 Å for 258 pairs of Cα atoms. As a member of the superfamily of short‐chain dehydrogenases/reductases (SDR),^[22]^ SrBDH1 folds into the characteristic Rossmann‐like fold,^[23]^ that harbors the cofactor NAD^+^, and has an additional short α‐helix at the C‐terminus (Figure 4, Figure S7). A closer inspection of both structures revealed remarkable differences in the cofactor binding sites. In the structure obtained from crystals under the high salt condition, we saw a defined electron density for the NAD^+^ cofactor in only one monomer (Figure S7). Notably, no NAD^+^ was added during protein purification or crystallization, therefore NAD^+^ was co‐purified with SrBDH1. Hence, the structure represents the binary complex (SrBDH1 ⋅ NAD^+^ /high salt). Binding of NAD^+^ leads to a defined folded loop region from residue V197 to S203. The well‐defined loop conformation might be a consequence of crystal packing, which hampers dissociation of the cofactor. The apo structure of SrBDH1 (Table S3) lacks electron density for NAD^+^, and the latter loop region is not defined. The remaining apo structure is practically indistinguishable from the structure of the binary complex.

In contrast, we do observe electron density for all four cofactor binding sites in the structure obtained under the PO/OH crystallization condition (SrBDH⋅NAD^+^/PO/OH) (Figure S7). The electron density clearly shows different sigma levels at the supposed binding groove for NAD^+^, indicating different occupancies of the NAD^+^ across the four monomers.

The active site is mainly lined by hydrophobic residues (V97, G99, I154, A155, F190, G191, I196, V197, and M211) and is composed of F260 from a symmetry‐related molecule (Figure 4C and D and Figure S7A). The NAD^+^, as well as the strictly conserved catalytic residues S146, Y159, and K163, complete the active site. For other studied SDRs, during oxidation, the hydroxyl group of the tyrosine abstracts a proton from the substrate. The adjacent lysine enhances the acid/base properties of the tyrosine, and the serine stabilizes and polarizes the carbonyl group of the substrate.^[24]^ Activity loss of the enzyme after substitution of S146 and Y159 to alanine confirmed their catalytic role.

Recently, the crystal structure of the unspecific PsBDH of *Pseudomonas* sp. TCU‐HL1 (PDB ID 6M5N) sharing 30 % of sequence identity with SrBDH1, was solved.^[25]^ The monomers of PsBDH and SrBDH1 ⋅ NAD^+^/high salt superimpose with a RMSD of 1.52 Å for 230 pairs of Cα atoms (Figure 4A). The largest differences between both structures are located in the last third of the amino acid sequence upstream of β‐strand βF (Figure 4A and B), affecting the putative borneol binding site. In the structure of SrBDH1, the β‐strand βF is connected by a single α‐helix αFG to αG, while in the structure of PsBDH we find an insertion of two α‐helices, αFG1 and αFG2. Moreover, SrBDH1 contains an additional C‐terminal α‐helix αH that is absent in PsBDH (Figure 4A, B, and C). Due to the differences in fold and secondary structure content, amino acids that are flanking the active site differ between both structures (Figure 4C, D, and E). Notably, the active site of SrBDH1 is further shielded by the α‐helix αH of a symmetry‐related molecule (Figure 4). The location of SrBDH1 αH is comparable to the α‐helix αFG2, which is more distant from the active site (Figure 4A and B), but from the identical monomer. Our observations are in accordance with previous publications stating that the C‐terminal portion generally functions in substrate binding, therefore, the obvious structural variation in this region results in the diversity of substrate specificities.^[26]^ A more detailed inspection of the active site revealed, that except for I154 of SrBDH1, none of the amino acids present the active site are conserved to PsBDH.

### Probing enantiospecificity by site‐directed mutagenesis

**Figure 5:**
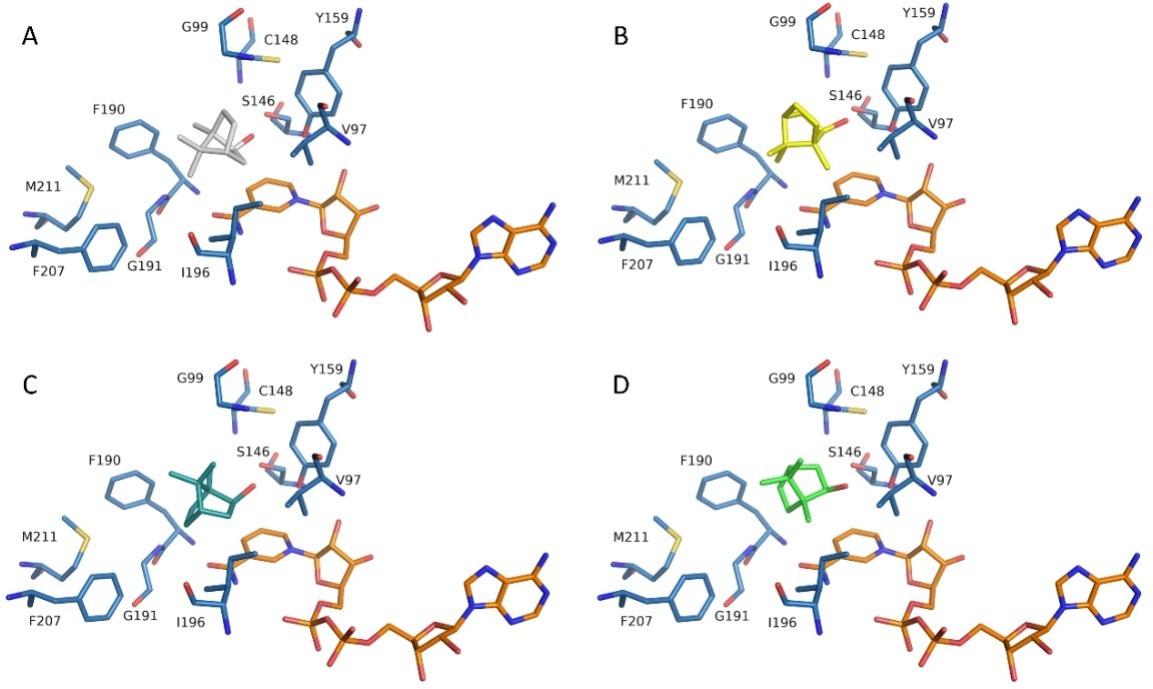
Docking results of (+)‐*endo*‐**1 a** (**A**), (−)‐*endo*‐**1 a** (manually docked, **B**), (+)‐*exo*‐**1 a** (**C**), (−)‐*exo*‐**1 a** (**D**) in the active site of SrBDH1. *Endo*‐**1 a** enantiomers seem to be located in the active site in a different orientation compared to *exo*‐**1 a** enantiomers, but the most striking difference is the methyl group that points away from the page in accepted molecules (**A** and **B**) whereas it is in the opposite direction in non‐accepted molecules (**B** and **D**).

Intrigued by these findings we proceeded to elucidate the origin of specificity in BDH‐type enzymes. Since soaking or co‐crystallization experiments with substrates failed in yielding a crystal structure with a bound substrate, we performed docking studies using both enantiomers of *endo*‐**1 a**, *exo*‐**1 a** and **1 b** to identify the most probable conformation in the active site. The bicyclic monoterpenols *endo*‐**1 a** and *exo*‐**1 a** are compact molecules that offer only small differences regarding asymmetries or polar groups that could facilitate stereodiscrimination by the enzyme. This was reflected in a large number of different poses (8–12) obtained in docking experiments (using a min RMSD of 2 Å for clustering). To identify productive binding modes, we lowered the clustering threshold to get more poses. We considered a pose to be productive when the hydrogen in α position from the hydroxyl group was located towards NAD^+^ and the hydrogen of the hydroxyl group was in range (d <4 Å) with the catalytic residues S146 and Y159 (Figure 5).^[24]^ The low ratio of productive binding mode is in agreement with the very high *K*_M_ of the enzyme. Docking of *exo*‐**1 a** yielded one productive pose for each enantiomer. In the case of *endo*‐**1 a**, the preferred (+)‐enantiomer led to a productive binding mode, whereas no productive pose was found for the (−)‐enantiomer. In this last case, we forced (−)‐*endo*‐**1 a** in a hypothetical binding mode in the crystallographic structure of the enzyme in order to study how we could enable its conversion (Figure 5B). When (+)‐*endo*‐**1 a** was soaked into crystals of SrBDH1 prior to structure determination, an additional patch of electron density (data not shown) was revealed. The volume and location of this additional electron density is in good agreement with the modeled position of (+)‐*endo*‐**1 a**. To further verify that the missing binding mode for (−)‐*endo*‐**1 a** might be possible, the same ligands were docked in the newly structurally characterized unselective BDH from *Pseudomonas* sp. TCU‐HL1 (PsBDH, PDB‐ID: 6M5N).^[23]^ In this case, a productive binding mode for (−)‐*endo*‐**1 a** could be found. The PsBDH seems to have more space in the hydrophobic pocket of the active site. However, the significantly different secondary structure in the active‐site pocket made it difficult to pinpoint concrete differences that could explain why productive poses for all ligands could be found in this dehydrogenase (Figure 4A and B).

The docking results highlighted two main potential differences between the positioning of the isomers. First, borneol and isoborneol seem to fit differently in the pocket. In particular, the two methyl groups in C7 assume a different position. Second, the methyl group in the chiral C1 in the binding modes of preferred (Figure 5A and C) and non‐preferred enantiomers (Figure 5B and D) points in opposite directions.

The crystallographic and docking studies served as a starting point for the selection of active site residues that might have a significant effect on stereospecificity. Furthermore, two strategies were combined to create variants with decreased specificity allowing us to identify residues that are responsible for the excellent enantiodiscrimination in SrBDH1 enzyme. The first strategy was to introduce residues found in the active site of non‐specific BDHs that differed from ones at the same positions in SrBDH1 (Table S4). The second strategy was to use the coupled moves protocol implemented in the Rosetta framework by Ollikainen et al.^[27]^ for the four isomers of **1 a**. The Rosetta protocol was designed to change substrate specificity by redesigning the active site of a certain enzyme. Specifically, for each isomer we obtained a set of positions in which certain residues could possibly have a positive impact on the binding energy of the enzyme for the isomer. We hypothesized that positions, where the enriched residues were different for each enantiomer, are important for the specificity of SrBDH1. The coupled moves highlighted positions such as V97F/Y, G99T/P, C148 A or I196 L (Figure S9). Table 4 shows the effect of amino acid substitutions in the active site on the specificity towards *endo*‐**1 a** and *exo*‐**1 a**. Mutagenesis points out the residues V97 and G191 as determinants of enantiospecificity for *exo*‐**1 a**. Valine 97 is located in direct vicinity to the cofactor and the substrate and is likely to exert an influence on substrate recognition. The coupled moves protocol suggested multiple mutations at this position, making us think that it might be an important site for enantiospecificity. However, the decision to substitute V97 by proline was made after observing that several non‐specific BDHs have a proline in the analogous position. Indeed, V97P has a reduced specificity (E=15), while conversions were similar to those of the wildtype enzyme. In homology models of the non‐specific AaADH2, the equivalent proline is in a loop that strongly differs from that of SrBDH1 (Figure S10). As V97P might have consequences on the protein backbone conformation of this loop, its effect on the enantiospecificity is not easy to rationalize. Glycine 191 is positioned at the opposite side of the active site (Figure 5) and its methylene group has a distance of 4 Å to both the nicotinamide moiety of the cofactor and the substrate. Exchanging G191F was done due to the presence of this residue in several non‐specific BDHs (Figure S1, Table S4) and led to a considerable reduction of activity towards *exo*‐**1 a**. F191 could undergo π‐stacking with F207 and lead to rearrangement of this part of the active‐site pocket. However, there is no obvious explanation why the V97P and G191F substitutions increase (−)‐*exo*‐**1 a** proportional formation, but do not affect the specificity for (−)‐*endo*‐**1 a**. Substitutions in M211 by valine and leucine showed a slight increase in the specificity for *exo*‐**1 a**, while maintaining the high specificity for *endo*‐**1 a**. M211 is located in an α‐helix that has not equivalent structure in PsBDH (Figure 4), at the opposite side of the active site where the hydroxyl group of the substrate is located (Figure 5).

**Table 4:** Conversion and enantiospecificity for the kinetic resolutions of *endo*‐**1 a** and *exo*‐**1 a** for the point mutants tested. The reaction mix contained 1 mM substrate, 1 mM of NAD^+^ and was incubated at 20 °C for 30 min before extraction for GC analysis.

		*endo* **‐1 a**				*exo*‐**1 a**		
								
Mutant		E^[a]^		[%]c^[a]^		E^[a]^		[%] c^[a]^
								
SrBDH1		>200		50 %		132		53 %
V97G^[b]^		>200		7 %		>200		4 %
V97C^[c]^		>200		50 %		47		45 %
V97P^[c],[b]^		>200		41 %		15		38 %
V97F^[d]^		>200		10 %		14		8 %
V97Y^[d]^		>200		4 %		>200		1 %
G99 N^[c]^		>200		37 %		>200		27 %
G99H^[c][e]^		n.c.^[f]^		n.c.		n.c.		n.c.
G99D^[c]^		>200		43 %		>200		38 %
G99T^[d]^		>200		50 %		76		51 %
C148A^[d]^		>200		50 %		>200		51 %
F190Y^[d]^		>200		47 %		>200		43 %
G191M^[c]^		>200		48 %		>200		49 %
G191S^[c]^		>200		17 %		>200		14 %
G191F^[c]^		>200		11 %		4		15 %
I196L^[c]^		>200		20 %		>200		14 %
F207W^[d]^		n.c.		n.c.		n.c.		n.c.
M211V^[b],[c]^		>200		50 %		>200		50 %
M211L^[c]^		>200		50 %		>200		50 %

[a] Calculated from ee_p_ and ee_s_ that were determined by chiral gas chromatography, [b] Selected according to docking results, [c] Selected according to sequence alignment analysis, [d] Selected according to results of Rosetta couple moves protocol, [e] Expressed in insoluble form, [f] n.c.=no conversion detected.

The mechanisms underlying the enantiospecificity of many enzymes are often characterized by well‐defined steric and electronic interactions. The different sizes of the two substituents of secondary alcohols guide the stereospecificity of lipases.^[28]^ Both (*S*)‐specific and (*R*)‐specific amine transaminases employ binding pockets for the accommodation of large and medium‐sized prochiral ketones, which often results in outstanding specificity.[^29^, ^30^] The stereoselectivity of ketoreductases in the asymmetric reduction of prochiral ketones follows the same principle.^[31]^ Many enzyme classes bind their substrates with a multitude of polar interactions, which allows them to discriminate between different polar groups on the substrate molecule;^[32]^ this principle allows carbohydrate‐converting enzymes a tremendous specificity toward molecules with several functional groups of similar reactivity.^[32]^ In contrast, the stereospecificity of plant SDR for bicyclic monoterpenols *endo‐* **1 a** and *exo*‐**1 a** is a curious case, as their carbon skeleton is rigid and does not have any rotational degrees of freedom. The compact bornane‐type structure does not show any obvious steric differences (such as a large and a small substituent) that would facilitate discrimination between both enantiomers. Possibly, *endo‐* **1 a** and *exo*‐**1 a** present minor steric differences in the accessibility of hydrogen in the chiral C1; in *exo*‐**1 a** this hydrogen is on the same plane as the methyl groups in C7 whereas in *exo*‐**1 a** it is on the less sterically‐crowded plane. This minor difference might explain the preference of most enzymes for *exo*‐**1 a** in the reverse reaction, here the hydride from NADH should access the less sterically‐crowded plane resulting in *exo*‐**1 a**. The positioning of the substrate can be deduced from the required short distances from C_1_‐H to the cofactor, and OH−H to S146 and Y159. Other than these, there are no clearly defined interactions between the substrate and the hydrophobic active site of SrBDH1 that could explain its very high specificity towards racemic *endo‐* **1 a** and *exo*‐**1 a**. SrBDH1 preferentially converts (+)‐*endo‐* **1 a** and (+)‐*exo*‐**1 a** and shows scarce activity towards the (−)‐enantiomers. Mutagenesis of 6 of the 8 residues of the active‐site pocket (the remaining two being highly conserved) led to the identification of two variants that additionally convert (−)‐*exo*‐**1 a**, but, not (−)‐*endo‐* **1 a**. This corresponds to the observation that in docking experiments, only (−)‐*endo‐* **1 a** did not form a productive binding mode. It is not clear whether the enantiospecificity towards *endo‐* **1 a**, the presumed natural substrate of these SDRs, has any evolutionary advantages for the plant. However, a possible benefit might be the possibility of specific oxidation of (+)‐*endo‐* **1 a** to (+)‐**2 a** in the presence of (−)‐*endo*‐**1 a**. Indeed, the essential oil of *R. officinalis* contains both (−)‐*endo‐* **1 a** and (−)‐*exo*‐**1 a**. In view of the very high enantiospecificity of *R. officinalis* towards borneol and the fact that its specificity was not affected by single‐site mutagenesis, it is indeed striking that a few of the investigated borneol‐dehydrogenases show specificity (Figure 1).

## Conclusion

A selected set of short‐chain borneol‐type dehydrogenases were characterized in this study in terms of substrate acceptance and specificity. All enzymes converting the substrates showed some extent of preference for (+)‐*endo*‐**1 a** and (+)‐*exo*‐**1 a** over their specular images, with the novel SrBDH2 presenting outstanding specificity for (+)‐*endo*‐**1 a** and SrBDH1 for both of them. The kinetic resolution of *endo*‐**1 a** utilizing SrBDH1 produces optically pure (+)‐**1 b** and allows the isolation of the unreacted (−)‐*endo*‐**1 a**. In the case of *exo*‐**1 a**, SrBDH1 yields (−)‐**1 b** and (−)‐*exo*‐**1 a** in optically pure form. The high activity, stability and specificity of SrBDH1, make this enzyme a promising biocatalyst for the preparation of optically pure *endo*‐**1 a**, *exo*‐**1 a**, (+)‐**1 b** and (−)‐**1 b**. Therefore, enzymatic catalysis utilizing SrBDH1 could substitute the currently used extraction from plants, which is unfavorable from both the environmental and economical point of view.

The distribution of enantiospecificity enzymes observed in the phylogenetic tree indicates that either the ability to oxidize borneol evolved independently several times during evolution or that a borneol oxidizing ancestor existed and some descendants lost the affinity for this substrate. Further characterization of different alcohol dehydrogenases belonging to SDR110 C group would be necessary in order to have a more complete and correct interpretation of the evolution of this family.

The structure of SrBDH1 showed a predominantly hydrophobic catalytic pocket. A comparison with the non‐specific PsBDH revealed major differences in the structure and amino acids shaping the active site pocket. Docking of the enantiomers of *endo*‐**1 a**, *exo*‐**1 a** and **1 b** was performed with SrBDH1 structure. These results displayed productive binding modes for (+)‐*endo*‐**1 a**, (+)‐*exo*‐**1 a** and (−)‐*exo*‐**1 a**, but not for (−)‐*endo*‐**1 a**. This is in agreement with the directed mutagenesis study, where the specificity for (+)‐*endo*‐**1 a** remained high for all mutations, whereas we could significantly reduce it for *exo*‐**1 a** with mutations at positions V97, G99 and G191. Despite the high structural similarity between *endo*‐**1 a** and *exo*‐**1 a**, SrBDH1 can discriminate between the diastereoisomers. In fact, (+)‐*endo*‐**1 a**, the supposed natural substrate, shares the same backbone with (−)‐*exo*‐**1 a**, still the found binding mode of (+)‐*endo*‐**1 a** would be unproductive for (−)‐*exo*‐**1 a**. Productive binding modes for both substrates seem to depend on different interactions making it difficult to alter the specificity with point mutations only. This study describes a curious case of enantiospecificity and shows that this feature, in the case of unfunctionalized molecules, can be achieved in nature relying only on multiple weak interactions. Based on our results, the specificity of borneol‐type SDRs appears to be more robust to point mutations compared to lipases, transaminases, ketoreductases or carbohydrate‐converting enzymes. A combinatorial mutagenesis approach such as CASTing could highlight hotspots with epistatic effects, which are extremely difficult to highlight with point mutations only. Nevertheless, the identification of a highly active, stable and specific SrBDH1 in a still untapped market niche nicely proofs once again the potential of biocatalytic applications.

## Experimental section

### Materials

All chemicals were bought from Sigma Aldrich (Germany), except for (+)‐*endo*‐**2 a** (1S,2R,4R) (AaBlocks, USA) and used without further purification. *E. coli* strain BL21 (DE3) was used for expression.

Protein and DNA concentration were measured using a Nanodrop 2000 UV‐Vis spectrophotometer (Thermo Scientific, USA) at 260 and 280 nm respectively. Absorbance at 340 and 550–800 nm was measured using an Eon plate reader (BioTek, USA). The enzymes were purified using ÄKTA pure system (GE Healthcare Life Sciences, Austria).

The genes were ordered at GeneScript (USA), codon‐optimized for *E. coli* and cloned into the vector pET15b in frame with an N‐terminal poly‐histidine tag.

### Phylogenetic tree and alignment

Evolutionary analyses were conducted in MEGA X.^[33]^ The evolutionary history was inferred by using the Maximum Likelihood method and Le_Gascuel_2008 model.^[34]^ The tree with the highest log likelihood is shown in Figure 1. Initial trees for the heuristic search were obtained automatically by applying Neighbor‐Join and BioNJ algorithms to a matrix of pairwise distances estimated using a JTT model, and then selecting the topology with superior log likelihood value. A discrete Gamma distribution was used to model evolutionary rate differences among sites (5 categories (+G, parameter=1.5189)). The rate variation model allowed for some sites to be evolutionarily invariable ([+I], 0.26 % sites). Evolview v2 was used for visualization.^[35]^

### Protein expression and purification

Protein expression and purification of AaADH2, PsBDH, SoBDH1 and SoBDH2 were done as described before.^[1]^ Briefly, for SrBDH1, SrBDH2 and SoBDH3, *E. coli* BL21 (DE3) chemo‐competent cells were transformed with the constructs. Overnight cultures of 12.5 mL were used to inoculate flasks of 500 mL of LB media supplemented with of ampicillin (100 mg L^−1^ final concentration). The flasks were shaken at 130 rpm at 37 °C for 3.5 h (until OD600 between 0.6‐0.8 was reached). The cultures were then induced with of IPTG (1 mM final concentration) and shaken overnight at 130 rpm at 28 °C.

Cells were harvested by centrifugation (*Beckman Coulter*, USA, JA10 rotor) at 6,000 rpm at 4 °C for 20 min. The pellets were resuspended in 20 mL of lysis buffer (20 mM Tris‐HCl, 500 mM NaCl, 1 mM DTT, 10 % glycerol, 20 mM imidazole, pH 8). The resuspended cells were sonicated for 6 min, output control 7, duty cycle 70 % and then centrifuged at 13,000 rpm for 45 min at 4 °C. The supernatants were recovered, filtered with 0.45 μm filters and loaded into pre‐equilibrated His‐Trap FF crude 5 mL columns (GE‐Healthcare, Austria) for affinity chromatography purification. The loaded columns were washed with 50 mL of binding buffer (20 mM Tris‐HCl, 500 mM NaCl, 10 % glycerol, 30 mM imidazole, pH 8). The purified enzymes were eluted using 30 mL of elution buffer (Tris‐HCl 20 mM, 500 mM NaCl, 10 % glycerol, 300 mM imidazole, pH 8) and dialyzed overnight with storage buffer (Tris‐HCl 20 mM, 500 mM NaCl, pH 8). If needed, the enzymes were concentrated using Amicon® Ultra 10 K centrifugal filter (Merck KGaA, Germany). The enzymes were aliquoted and stored with a final concentration of 10 % glycerol at −20 °C. Following the purification, the fractions were analyzed by SDS‐PAGE electrophoresis on 12 % polyacrylamide gels (ExpressPlus™ PAGE Gel, Genscript, USA) followed by staying with Coomassie Brilliant Blue. Protein concentration was determined by measuring absorbance at 280 nm. The extinction coefficients for the enzymes were obtained from ExPASy ProtParam Tool (https://web.expasy.org/protparam/). The ϵ_280_ for the monomers of the enzymes correspond to 9,190 M^−1^ cm^−1^ for SrBDH1, 10,680 M^−1^ cm^−1^ for SrBDH2, and 13,200 M^−1^ cm^−1^ for SoBDH3.

### Size exclusion chromatography

Size exclusion chromatography was carried using a HiLoad™ 16/60 200 Superdex™ column (GE Healthcare). The column was equilibrated overnight with buffer Tris‐HCl 100 mM, 500 mM of NaCl, pH 8. 500 μL samples were injected and then eluted at a flow of 1 mL min^−1^ in 1.5 column volumes. A calibration curve was elaborated using Gel Filtration Cal Kit High Molecular Weight (GE Healthcare). The molecular mass standards used were Ovalbumin (44 kDa), Conalbumin (75 kDa) Aldolase (158 kDa) and Ferritin (440 kDa).

### Determination of conversion, enantiomeric excess and E value for the oxidative and reductive reactions

For the oxidations, a reaction volume of 1 mL containing the alcohol dehydrogenase (15 μM of the monomer of AaADH2, PsBDH, SoBDH1, SoBDH2, SoBDH3, SrBDH1, SrBDH2), 1 mM of NAD^+^, 1 mM of substrate (*endo*‐**1 a**, *exo*‐**1 a**, *exo*‐**2 a** or *endo*‐**3 a**) in buffer Tris‐HCl 100 mM, 500 mM of NaCl, pH 8 at 20 °C, 600 rpm was used. Samples of 200 μL were taken at different time points for gas chromatography analysis. All the reactions were done in duplicate.

A colorimetric screening based in phosphate detection^[36]^ was used to identify activity for the reductive reaction of the enzymes. The reactions were prepared in a 96 deep well plate by triplicate. The reaction mix of 1 mL consisted in 15 μM (monomer) of the alcohol dehydrogenase determined by the absorption at 280 nm (AaADH2, PsBDH, SoBDH1, SoBDH2, SoBDH3, SrBDH1 or SrBDH2), 20 μM of NADH, 10 mM of sodium phosphite, 12 μM of phosphite dehydrogenase and 5 mM of substrate (**1 b**, **2 b** or **3 b**) in citrate buffer (50 mM, 500 mM NaCl, pH 5.5). The reactions were incubated at 20 °C, 200 rpm for 48 h. Samples of 200 μL were taken at 0, 24 and 48 h and frozen with liquid nitrogen. For phosphate measurement, a mix of 200 μL of molybdate reagent ((CH_3_CO_2_)_2_Zn 100 mM, ammonium molybdate ((NH_4_)_6_Mo_7_O_4_.4H_2_O) 10 mM, pH 5 adjusted with HCl), 50 μL of ascorbic acid solution (L‐(+)‐ ascorbic acid 10 %, pH 5 adjusted with NaOH) and 20 μL of sample was put together in a 96 well plate. After 30 min at 37 °C, absorbance in the range between 550–800 nm was measured. A calibration curve with known phosphate concentrations from 0 to 10 mM (triplicate) was used for interpretation of the results. The reactions that after 48 h showed some level of conversion were extracted and analyzed by GC‐FID.

### Determination of kinetic parameters and specific activities

Kinetic parameters were determined measuring NADH formation at 340 nm. Reaction mixes were prepared in 96 well plates for UV measurement by triplicate. For (+)‐*endo*‐**1 a** kinetic study: 0.5 μM of SrBDH1 (tetramer), 1 mM NAD^+^, (+)‐*endo*‐**1 a** in a range between 0.5 and 6 mM, 5 % DMSO, buffer Tris‐HCl 100 mM pH 9. For NAD^+^ kinetic study: 0.5 μM of SrBDH1, NAD^+^ in a range between 0.05 and 2 mM, 5 mM of (+)‐*endo*‐**1 a**, 5 % DMSO, buffer Tris‐HCl 100 mM pH 9. All the reactions were started adding NAD^+^ to the reaction mix. Absorbance at 340 nm was measured every 15 s for 30 min. The linear range of the curves was used to calculate the initial rates. Origin 2019b (OriginLab Corporation, USA) was used for the nonlinear fitting using Michaelis‐Menten model to obtain the kinetic parameters.

Specific activities were obtained in a similar way under the following conditions: 20 μM of the alcohol dehydrogenase (monomer) (AaADH2, PsBDH, SoBDH2, SoBDH3, SrBDH1 or SrBDH2), 2 mM NAD^+^, 2 mM of substrate, 1 % DMSO in Tris‐HCl 100 mM pH 9.

### Optimum pH determination for SrBDH1

SrBDH1 was incubated for 30 min on the following buffers: citrate buffer 100 mM for pH 4.5, 5 and 5.5; potassium phosphate buffer 100 mM for pH 6, 6.5 and 7; Tris‐HCl buffer 100 mM for pH 7.5, 8, 8.5 and 9; carbonate‐bicarbonate buffer 100 mM for pH 9.5, 10 and 10.5. After that time, 5 μL of the incubated enzyme were added to a mix consisting in 4 mM of (+)‐*endo*‐**1 a**, 5 % DMSO, 5 mM NAD^+^, 0.15 μM of SrBDH1 in buffer Tris‐HCl 100 mM pH 9 in a final volume of 200 μL. Absorbance at 340 nm was measured every 15 seconds for 45 min. The linear range of the curves was used to calculate the initial rates of reaction. All the reactions were done by triplicate.

### Gas Chromatography‐flame ionization detector (GC‐FID) analysis

GC‐FID analysis was carried using a Shimadzu QP2010 SE GC‐FID system. All extractions were performed using 400 μL of DCM and 200 μL of sample at 0, 24 and 48 h. After mixing and discarding the inorganic phase, the samples were dried using Na_2_SO_4_, centrifuged and transferred to 1.5 mL vials with 200 μL inserts. For **1 a**, **1 b**, *exo*‐**2 a** and **2 b**, an Hydrodex β‐6TDM chiral column (Macharey‐Nagel) (25 m, 0.25 mm I.D., 0.25 μM film thickness) was used with the following program: 60 °C for 8 min, a linear increase of 2 °C/min to 150 °C, a linear increase of 40 °C/min to 200 °C, 200 °C for 2 min. For *endo*‐**3 a** and **3 b**, an Hydrodex‐β‐6TBDAc chiral column (Macharey‐Nagel) (50 m, 0.25 mm I.D., 0.25 μM film thickness) was used with the following program: 50 °C for 15 min, a linear increase of 1 °C/min to 110 °C, a linear increase of 20 °C/min to 220 °C, 220 °C for 1 min.

### Protein expression and protein purification for crystallization experiments

*E. coli* BL21‐RIL was transformed with pET15a vector containing SrBDH1 fused to an N‐terminal hexa‐histidine‐tag. Protein induction was carried in auto‐induction media at 37 °C for 7 h and subsequently cooled down to 16 °C.^[37]^ Cells were grown over night and harvested by centrifugation (10 min, 7,000 rpm at 4 °C). The pellets were resuspended with buffer A (20 mM Tris/HCl pH 8.0, 500 mM NaCl). Cells were lysed by homogenization at 4 °C for 7 min after addition of 0.5 mg l^−1^ DNase and the lysate was cleared by centrifugation (30 min, 21’500 rpm at 4 °C). Ni^2+^‐NTA beads (cv 1 ml, GE Healthcare) were equilibrated with buffer A. SrBDH1 was loaded on the column and washed with 15 cv of buffer A. SrBDH1 was eluted using a linear gradient with increasing imidazole concentration up to 300 mM. Size exclusion chromatography (SEC) was performed with a HighLoad Superdex S200 16/60 column (GE Healthcare), equilibrated with buffer B (20 mM Tris/HCl, pH 8.0, 125 mM NaCl). Pooled protein fractions were concentrated with Amicon‐Ultra‐15 (Merck KGaA) to 27.3 mg ml^−1^ as measured by the absorbance at 280 nm.

### Crystallization

Crystals were obtained by the sitting‐drop vapor‐diffusion method at 18 °C with a reservoir solution composed of 0.1 M Bis‐Tris/HCl pH 5.5 to pH 7.2, and NaCl ranging from 2.7 M to 3.2 M. Crystals were cryo‐protected with 25 % (v/v) glycerol supplemented to the reservoir resolution and subsequently flash‐cooled in liquid nitrogen. A second crystallization condition was obtained with a reservoir solution composed of 0.1 M HEPES/NaOH pH 7.0 to pH 7.8, 5/4 pentaerythritol propoxylate (PO/OH) 25 % (v/v), 30 %, 35 %, 0.1 M and 0.2 M KCl. Crystals were cryo‐protected with 15 % (v/v) glycerol supplemented to the reservoir resolution and subsequently flash‐cooled in liquid nitrogen.

### Soaking and co‐crystallization experiments

Crystals were soaked for 70 min in (+)‐borneol (80 mM from 400 mM stock in 100 % DMSO) containing cryo‐protectant and subsequently flash‐cooled in liquid nitrogen. For co‐crystallization 20 mM (+)‐borneol (from 100 mM stock in 100 % ethanol) were added to the protein solution and incubated for 1 h on ice prior crystallization. Crystals were cryo‐protected as above and subsequently flash‐cooled in liquid nitrogen.

### Diffraction data collection, structure determination and refinement

Synchrotron diffraction data were collected at the beamline 14.1 and 14.2 of the MX Joint Berlin laboratory at BESSY (Berlin, Germany). X‐ray data collection was performed at 100 K. Diffraction data were processed with XDS^[38]^ (Table S3). The structure for the SrBDH1 apo was solved via molecular replacement in PHASER^[39]^ by using the structure of the ternary‐secoisolariciresinol dehydrogenase from *Podophyllum petatum* (PDB ID 2bgm^[40]^) as search model. Crystals of SrBDH1 apo belong to the space group *P*4_1_2_1_2, with two molecules in the asymmetric unit. Model building and water picking were performed with COOT.^[41]^ The structure was initially refined by applying a simulated annealing protocol and in later refinement cycles by maximum‐likelihood restrained refinement using PHENIX.refine.[^42^, ^43^] The crystals of SrBDH1 NAD^+^ crystallized in space group *P*6_5_ and the structure was solved by molecular replacement with the SrBDH1 structure, computed from the crystals of the high salt condition, as search model. Model quality was evaluated with MolProbity^[44]^ and the JCSG validation server.^[45]^ Figures were prepared using PyMOL (Schrödinger, Inc). Secondary structure elements were assigned with DSSP,^[46]^ and ALSCRIPT^[47]^ was used for secondary structure‐based sequence alignments. Structure factor amplitudes and coordinates have been deposited in the ProteinDataBank.

### Multi‐angle light scattering (MALS)

MALS experiment was performed at 18 °C. SrBDH1 was loaded onto a Superdex 200 increase 10/300 column (GE Healthcare) that was coupled to a miniDAWN TREOS three‐angle light scattering detector (Wyatt Technology) in combination with a RefractoMax520 refractive index detector. For calculation of the molecular mass, protein concentrations were determined from the differential refractive index with a specific refractive index increment (*d*n/*d*c) of 0.185 ml g^−1^. Data were analyzed with the ASTRA 6.1.4.25 software (Wyatt Technology).

### Docking studies

(+)‐*endo*‐**1 a**, (−)‐*endo*‐**1 a**, (+)‐*exo*‐**1 a**, (−)‐*exo*‐**1 a**, (+)‐**1 b** and (−)‐**1 b** were energy minimized using a MM2‐force field for molecular docking into the active site of a monomeric representation of SrBDH1 and PsBDH (PDB‐ID: 6M5N). The docking was performed using the AutoDockVina program environment of YASARA Structure.[^48^, ^49^] All 6 substrate structures were docked into a simulation cell (X size=16 Å, Y size=16 Å, Z size=16 Å; angles: α=90°, β=90°, γ=90°) extended 1 Å around the residues I96, I154, G195 and I210 in SrBDH1 and the corresponding residues V95, I152, M193 and A208 in PsBDH. For each substrate, 999 docking runs were performed with atoms and bonds of the corresponding substrates set as flexible. Docking of each substrate resulted in one or more clusters using a rmsd cutoff for clustering of 2.0 Å.

### Coupled moves

The SrBDH1 structure was pre‐processed by running the Relax protocol with (+)‐*endo*‐**1 a** in the active site to minimize artefacts in the following protocol. The ligand rotamer library was generated by the free online tool Frog2^[50]^ with default options. The coupled moves protocol was run according to published setting^[27]^ (command line) as reported in the Supplementary Information.

The resulting output sequences were filtered for redundancy and analyzed using the script analyze_coupled_moves.py made available by the Kortemme lab in github.com^[51]^ which allows highlighting of the top 10 mutations enriched in the ligand of interest compared to a known accepted ligand and furthermore it allows the visualization of results via WebLogo.^[52]^ In all cases the Rosetta version 3.11 was used.

### Point Mutations

The QuikChange method (Agilent Technologies) was used for site‐directed mutagenesis. For the complete list of the primers used, please refer to the Supplementary Table S5). After PCR for the insertion of the mutants in pET15b_SrBDH1 plasmid, 10 U *Dpn*I were added to the mix and incubated at 37 °C for 1 h. Chemocompetent *E. coli* Top 10 cells were transformed using 3 μL of the mix. The mutants were confirmed by sequencing and retransformed in *E. coli* BL21 (DE3).

Cell‐free extract of SrBDH1 mutants was obtained as described before for the wild type. For biotransformations, 1 mM of *endo*‐**1 a** or *exo*‐**1 a**, 1 mM of NAD^+^ and 975 μL of cell‐free extract were used, for a total reaction volume of 1 mL. Samples of the reaction after 30 min and 24 h were extracted for chiral GC‐analysis.

### Author Contributions

R.K., B.L., A.C. and I.D. conceived the project and main conceptual ideas. A.C., N.D., M.P.P. and C.P.O.H carried out experiments, analyzed results and prepared figures for the paper. E.C. performed molecular modeling. E.C., L.P.P., M.H. and V.S. participated in the analysis of the results. All authors discussed the results and participated in the writing of the manuscript.

## Funding Sources

The authors acknowledge financial support by the German Federal Ministry of Education and Research (BMBF) for the project CbP‐camphor based polymers within the bio‐economy international program (grant No. 031B050B). R.K. and A.C. also would like to thank the Austrian Science Funds (FWF, P31001‐B29) for financial support. C.P.H. is supported by the Hanns Seidel Foundation.

## Abbreviations

SDR: Short‐chain dehydrogenase‐reductase
SrBDH1: Borneol‐type dehydrogenase 1 from *Salvia Rosmarinus*
SrBDH2: Borneol‐type dehydrogenase 2 from *Salvia Rosmarinus*
SoBDH3: Borneol‐type dehydrogenase 3 from *Salvia officinalis*
SoBDH1: Borneol‐type dehydrogenase 1 from *Salvia officinalis*
SoBDH2: Borneol‐type dehydrogenase 2 from *Salvia officinalis*
PsBDH: Borneol dehydrogenase from *Pseudomonas* sp. Strain TCU‐HL1
AaADH2: Alcohol dehydrogenase 2 from *Artemisia annua*
CCR2: CC chemokine receptor 2
CCL2: CC chemokine ligand 2
CCR5: CC chemokine receptor 5
TLC: thin‐layer chromatography

## Conflict of interest

There are no conflicts to declare.

## Supporting information

As a service to our authors and readers, this journal provides supporting information supplied by the authors. Such materials are peer reviewed and may be re‐organized for online delivery, but are not copy‐edited or typeset. Technical support issues arising from supporting information (other than missing files) should be addressed to the authors.

Supplementary
