# Supplementary material for: A Structural View on the Stereospecificity of Plant Borneol‐Type Dehydrogenases
Source: ChemCatChem. 2021 Mar 10;13(9):2262–77. doi: 10.1002/cctc.202100110 (PMC8261865; doi:10.1002/cctc.202100110)
Supplement: Supplementary file 1 — Supplementary [file CCTC-13-2262-s001.pdf]

ChemCatChem

Supporting Information

A Structural View on the Stereospecificity of Plant Borneol-Type Dehydrogenases

Andrea M. Chánique[+], Nicole Dimos[+], Ivana Drienovská, Elia Calderini, Mónica P. Pantín, Carl P. O. Helmer, Michael Hofer, Volker Sieber, Loreto P. Parra, Bernhard Loll,* and Robert Kourist*

Index

1. Supplementary Figures 3
Figure S1. 3
Figure S2. 4
Figure S3. 5
Figure S4. 6
Figure S5. 7
Figure S6: 7
Figure S7 8
Figure S8. 9
Figure S9. 9
Figure S10. 10
2. Supplementary tables 11
Table S1. 11
Table S2. 12
Table S3. 13
Table S4. 14
Table S5. 15
3. Nucleotide and amino acid sequences of the enzymes used in this study 16
4. Complete name and accession numbers of SDR used in phylogenetic tree 19
5. Commands used for couple moves protocol 21
References 22

1. Supplementary Figures

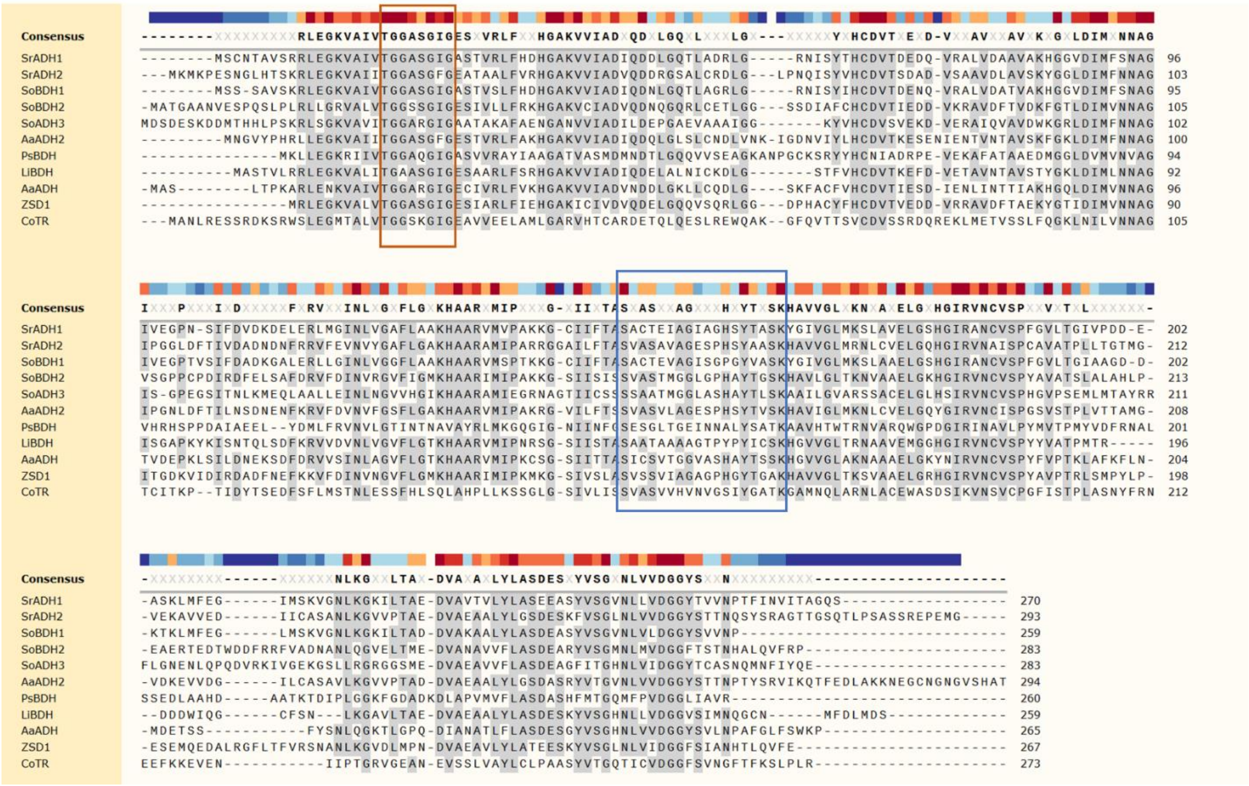

Figure S1. Alignment of new amino acid sequences from *Salvia officinalis*, *Salvia rosmarinus* and enzymes previously described to catalyze the oxidation of borneol. The sequences were aligned using Mega X with parameters defined by default and visualized in SnapGene 5.1. Residues matching the consensus sequence are highlighted in grey. Degree of conservation is indicated on top, with blue representing the less conserved residues and red the most conserved ones. "Classical" SDR motif for cofactor binding (TGxxx[AG]xG) is highlighted in an orange square. The catalytic triad (S, Y, K), including the active site "classical" SDR motif (YxxxK) is highlighted in a blue square.

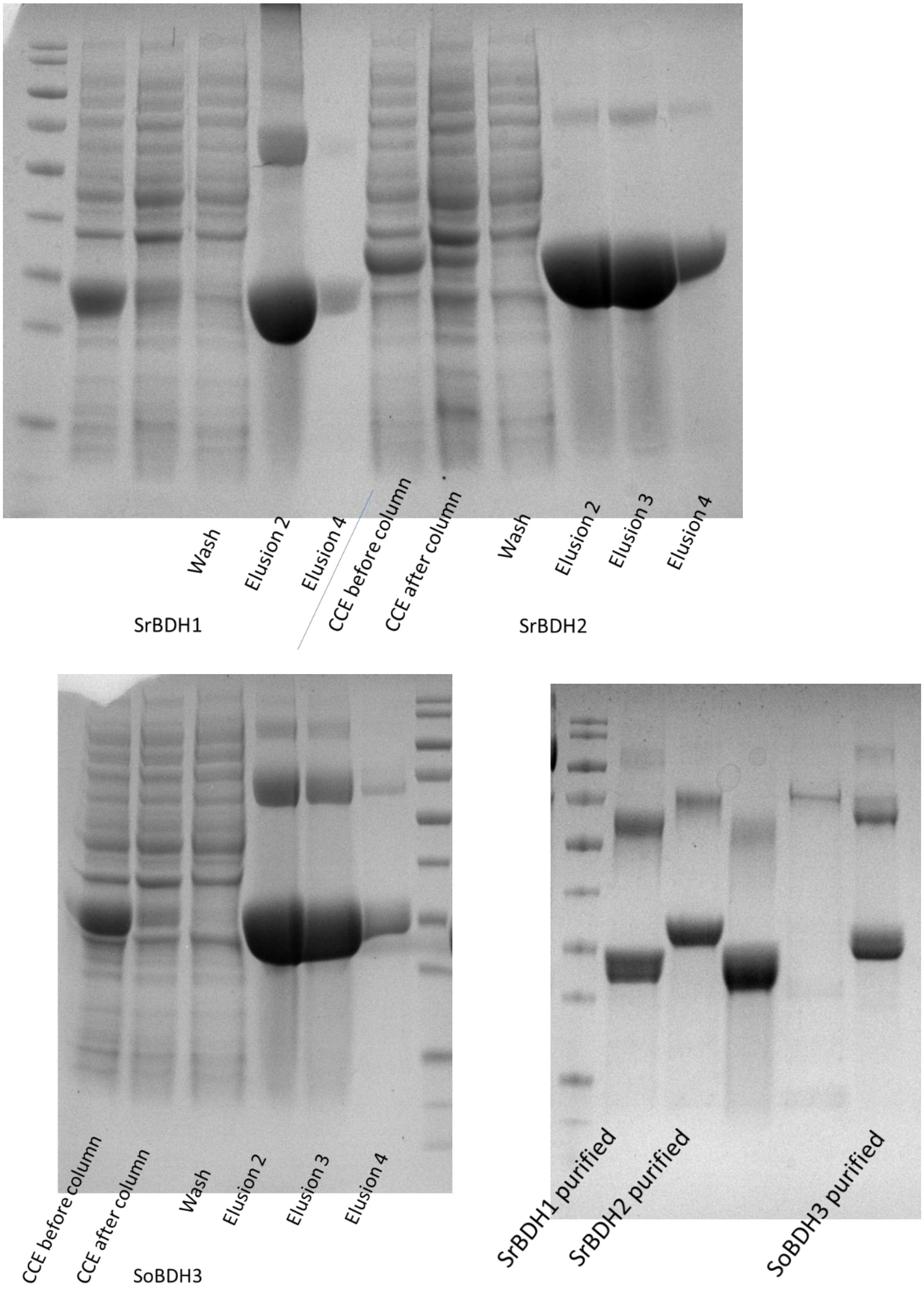

Figure S2. SDS gels for the purification of the three new BDH. CFE: cell free extract.

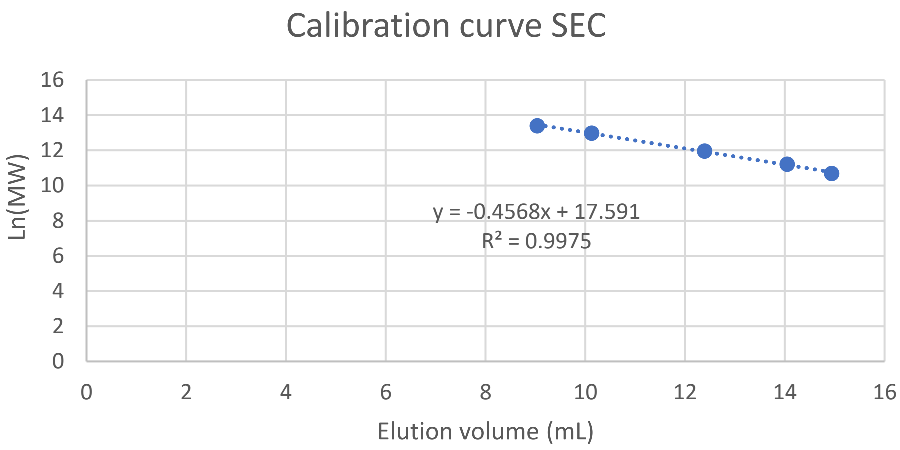

	Retention time (min)	Molecular weight according to calibration curve (Da)	Theorical molecular weight of the monomer (Da)	Oligomeric state
**SrBDH1**	12.98242	115917.1	30247.53	3.8
**SrBDH2**	12.392845	151743.7	32395.52	4.7
**SrBDH3**	12.557154	140771.3	31812.02	4.4

Figure S3. Graph showing calibration curve for size exclusion chromatography and table with the oligomeric state of SrBDH1, SrBDH2 and SoBDH3.

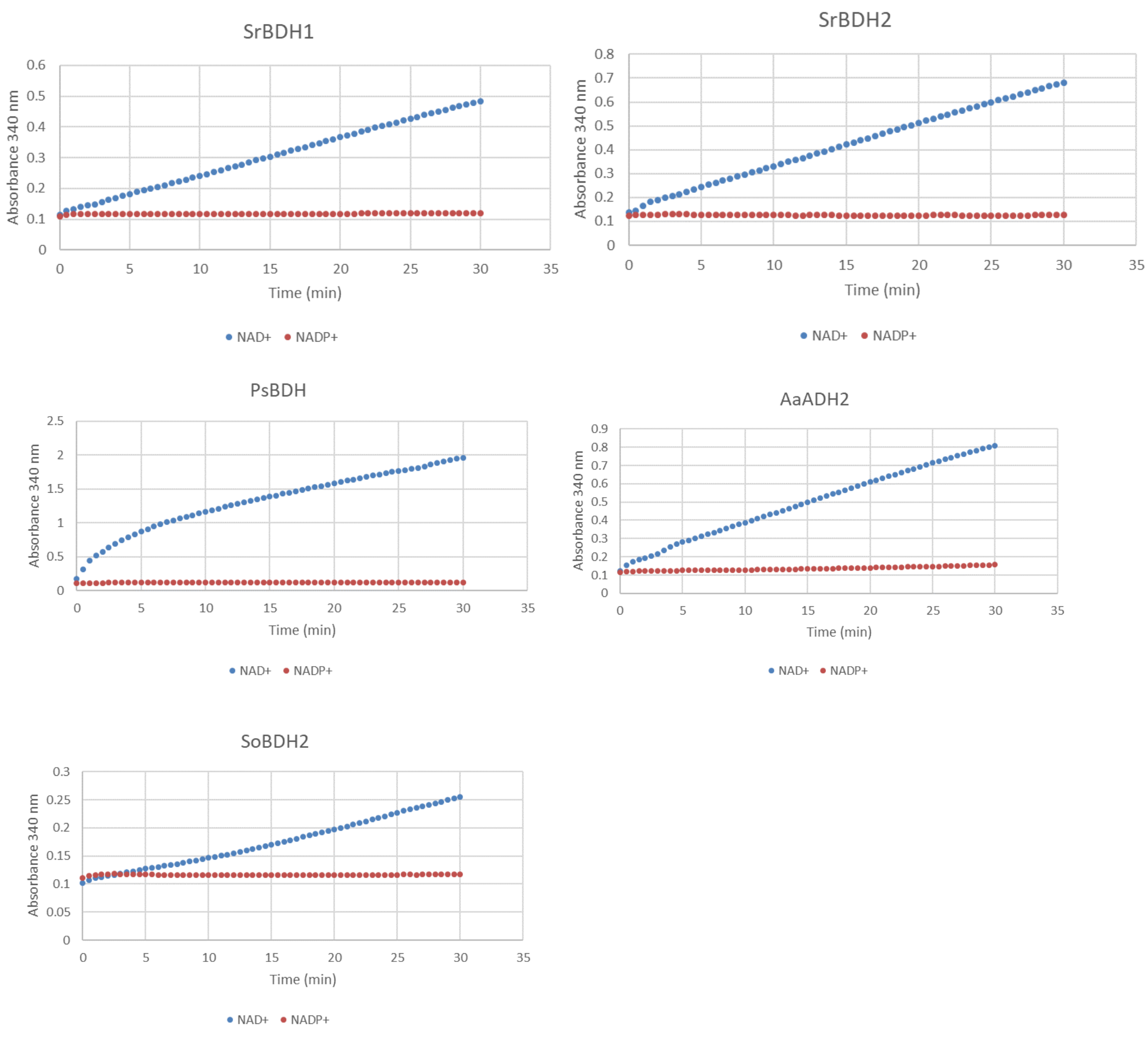

Figure S4. Graphs showing absorbance at 340 nm for NAD^+ and $NADP^+$ for borneol dehydrogenase-like enzymes. 2 µM of AaADH2, PsBDH, SoBDH2 and SrBDH1 or 20 µM of SrBDH2, 2 mM $NAD(P)^+$, 2 mM *exo*-**1a**, 1% DMSO, buffer Tris-HCl 100 mM pH 9.

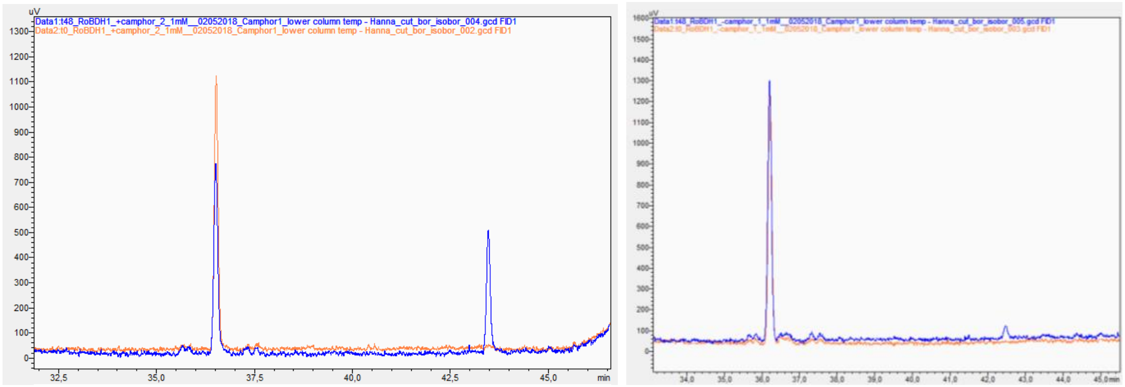

Figure S5. Chromatograms for conversion of pure enantiomers (+)-**1b** (left) and (-)-**1b** (right) catalyzed by SrBDH1. Orange for time 0 and blue for 48 hours.

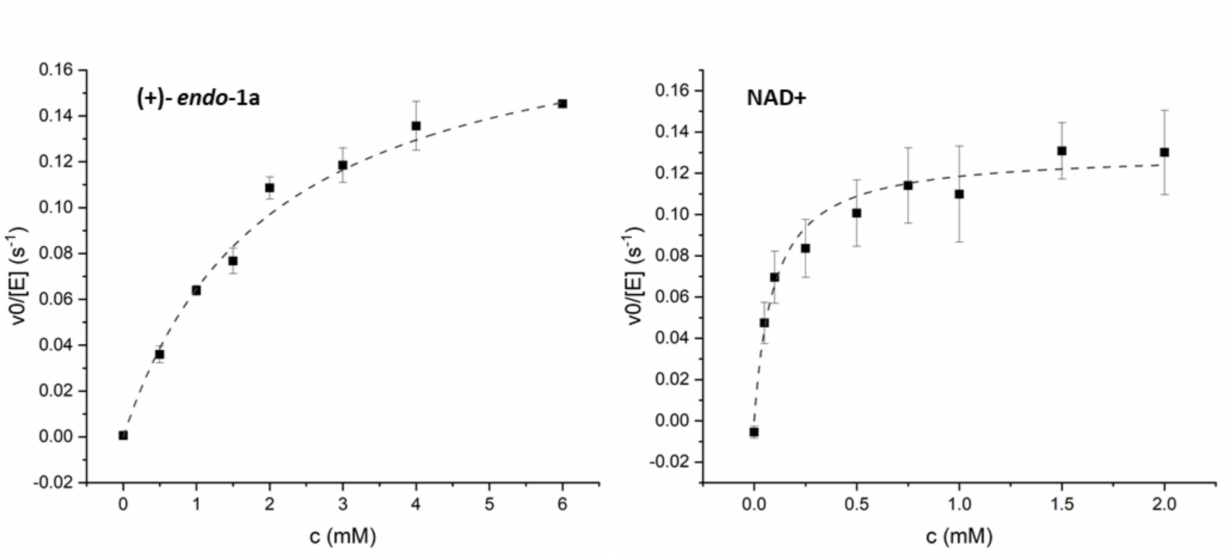

k_{cat} (s^{-1})	K_M (mM)	k_{cat}/K_M ($M^{-1}\ s^{-1}$)
0.20 ± 0.01	2.02 ± 0.18	96.53

k_{cat} (s^{-1})	K_M (μM)	k_{cat}/K_M ($M^{-1}\ s^{-1}$)
0.13 ± 0.01	96.99 ± 26.49	1,340.35

Figure S6: Kinetic characterization of SrBDH1 for (+)-*endo*-**1a** (left) and NAD^+. The lines represent the fit obtained using the Michaelis-Menten equation constructed using Origin 2019 software.

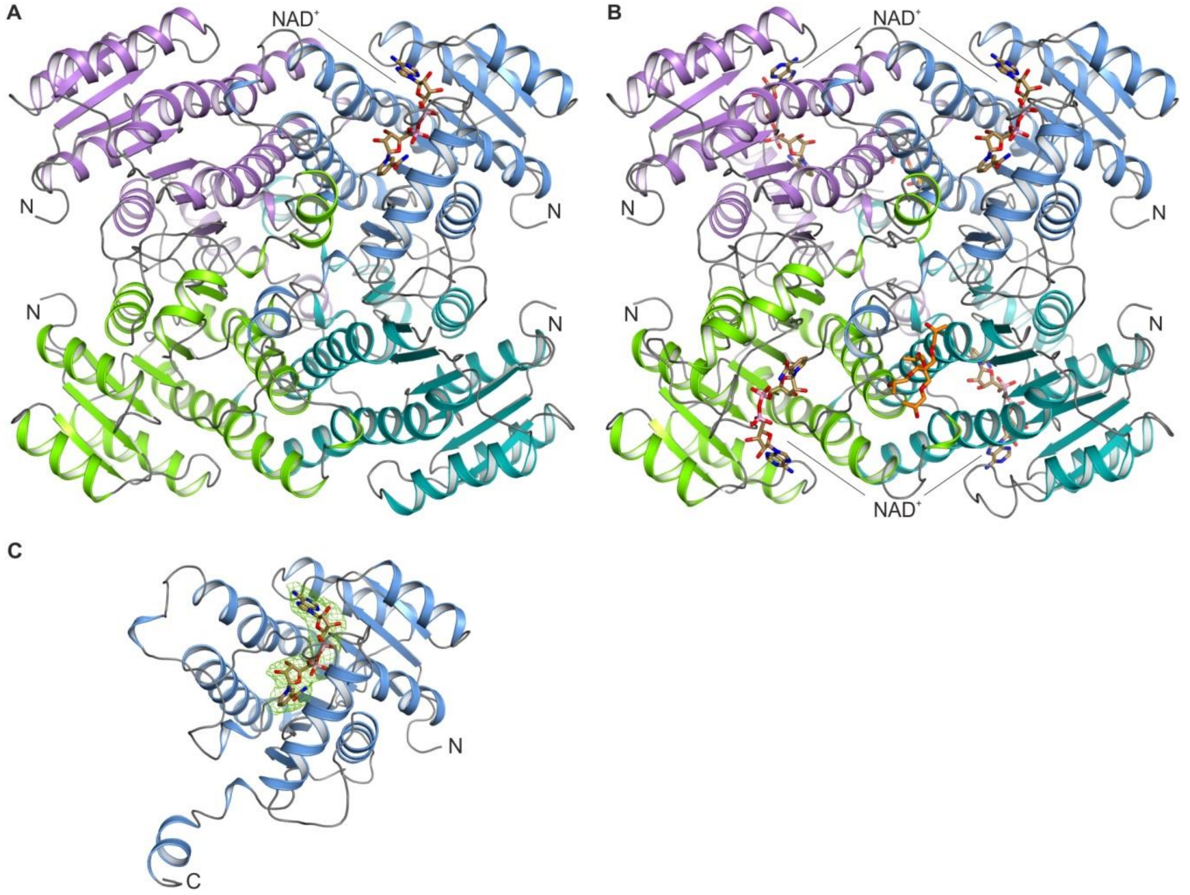

Figure S7. **(A)** Overall architecture of SrBDH1•NAD$^+$ / high salt forming a tetramer shown in cartoon representation. Only one NAD$^+$ molecule is bound to one monomer (chain C) depicted in stick representation. **(B)** Overall architecture of SrBDH1•NAD$^+$ / PO/OH. Identical view as in panel (A). NAD$^+$ is shown in brown stick representation. Quality of the electron density clearly indicates that the cofactor is not fully occupied. Two molecules of PO/OH are clearly defined in the electron density shown in orange stick representation. **(C)** Some view as in panel (A) but merely of the protein chain C in blue cartoon representation with the bound NAD$^+$. A POLDER map[1] was calculated for the omitted NAD$^+$. The electron density map is shown as green mesh contoured at 3 σ.

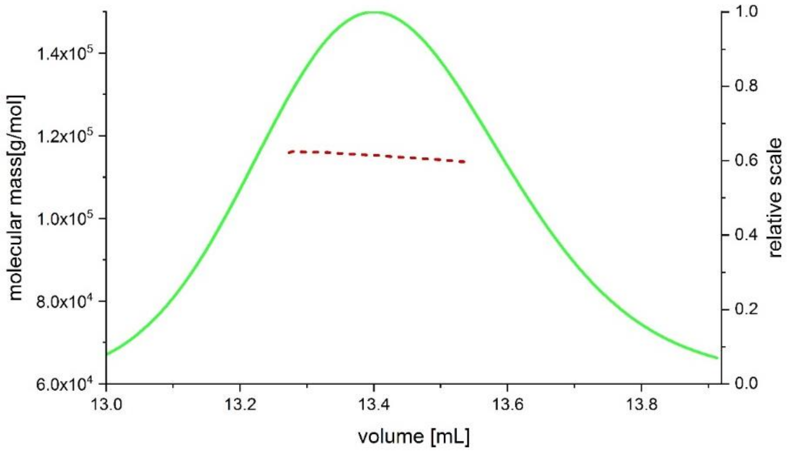

Figure S8. SEC/MALS analysis of SrBDH1. Solid, green curve represents the refractive index trace and the red curve the molecular mass at the corresponding elution volumes.

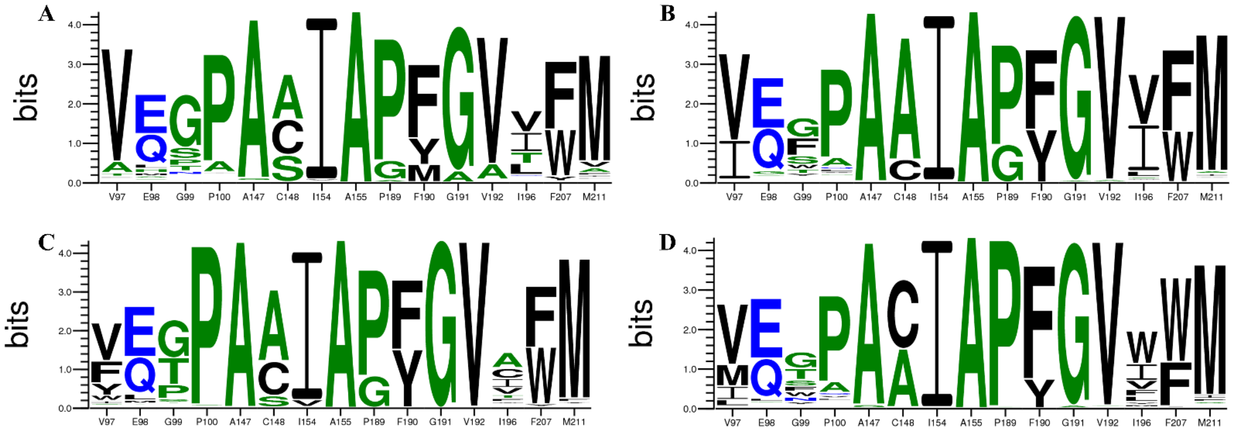

Figure S9. Residues suggested by the coupled moves protocol implemented in the Rosetta framework by Ollikainen et al.[2] for a better fit in the active site of (+)-*endo*-**1a** (A), (+)-*exo*-**1a** (B), (-)-*endo*-**1a** (C) and (-)-*exo*-**1a** (D).

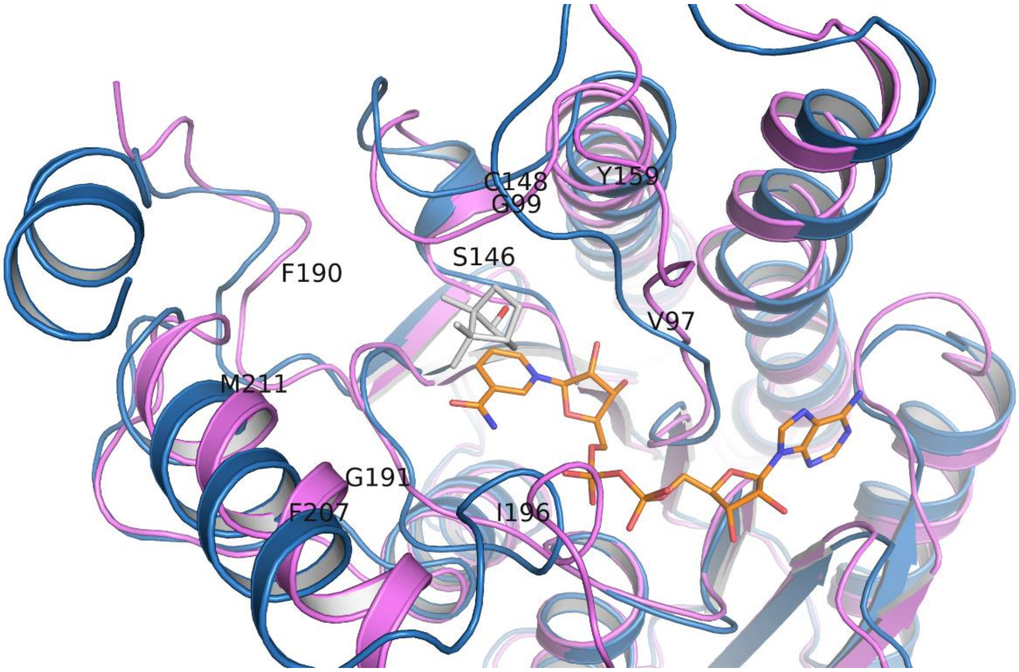

Figure S10. Secondary structure of the active site of SrBDH1 (blue) and AaADH2 (homology model) (violet). Positions mutated are labeled for SrBDH1. (+)-*endo*-**1a** showed in white and NAD^+ in orange.

2. Supplementary tables

Table S1. Specific activities measured for AaADH2: alcohol dehydrogenase from *A. annua*[3]; SoBDH2: borneol dehydrogenase from *S. officinalis L.*[4]; SrBDH1/2: borneol- like dehydrogenases from *S. rosmarinus*; SoBDH3: borneol-like dehydrogenase from *S. officinalis L*. PsBDH: borneol dehydrogenase from *Pseudomonas sp.* TCU-HL1.[5] The highest activity for each enzyme is highlighted in bold letters. Reactions with substrates displaying specific activities less than twice the blank are not shown. [a] n.c. = no conversion detected.

	Specific activity (mU/mg)					
**Substrate**	**SrBDH1**	**SrBDH2**	**SoBDH3**	**AaADH2**	**SoBDH2**	**PsBDH**
endo-**1a**	**30.22±0.46**	**9.98±0.46**	n.c.	**21.52±0.77**	**17.92±0.20**	**122.12±21.54**
exo-**1a**	23.66±0.36	5.05±1.71	0.30±0.05	53.34±1.29	12.74±0.56	114.78±5.17
exo-**2a**	4.80±0.04	1.54±0.13	n.c.	29.29±1.44	n.c.	68.59±3.58
(+)-*endo*-**2a**	3.92±0.36	5.89±1.04	0.27±0.04	22.50±0.81	n.d.	28.12±0.63
endo-**3a**	4.63±0.22	1.03±0.04	n.c.	14.48±0.53	7.05±1.03	29.54±1.86
**4a**	2.88±0.12	15.89±1.09	0.46±0.03	87.80±2.73	12.25±1.44	5.02±1.55
**5a**	3.33±0.07	n.c.	n.c.	29.10±2.87	6.69±0.78	28.49±3.12
**6a**	10.01±0.15	2.52±0.18	n.c.	16.76±0.73	18.03±0.0.46	22.81±1.46
**7a**	4.40±0.62	n.c.	0.23±0.02	20.54±0.47	38.79±2.94	17.51±0.83
**8a**	8.34±0.32	n.c.	0.21±0.11	14.75±2.11	3.76±0.25	10.69±0.66
**9a**	n.c.	n.c.	n.c.	n.c.	n.c.	n.c.
**10a**	n.c.	n.c.	n.c.	5.94±0.36	n.c.	n.c.
**11a**	n.c.	n.c.	n.c.	n.c.	n.c.	n.c.
**12a**	12.93±1.20	4.36±0.82	n.c.	8.70±5.17	2.26±0.10	42.98±5.8
**13a**	9.75±0.44	1.98±0.10	n.c.	62.73±2.74	6.33±0.13	6.15±0.12
**14a**	n.c.	n.c.	n.c.	9.96±1.97	n.c.	n.c.
**15a**	n.c.	n.c.	n.c.	n.c.	3.36±0.11	n.c.
**16a**	n.c.	n.c.	n.c.	4.77±0.34	4.72±0.54	n.c.
**17a**	3.27±0.14	-	0.20±0.01	-	6.45±0.49	3.24±0.18

Table S2. Conversions for (+)-**1b** and (-)-**1b** at 24 and 48 h with different concentrations of substrate.

(+) camphor	1 mM	2 mM	5 mM
24 h	22%	24%	51%
48 h	39%	45%	81%

(-) camphor	1 mM	2 mM	5 mM
24 h	7%	6%	6%
48 h	9%	10%	11%

Table S3. Crystallographic data

Data collection			
	SrBDH1•NAD$^+$ / high salt	SrBDH1•NAD$^+$ / PO/OH	SrBDH1 apo / high salt
Wavelength [Å]	0.9184	0.9184	1.0332
Space group	*P*4$_3$2$_1$2	*P*6$_5$	*P*4$_3$2$_1$2
Unit cell parameters<br>a, b, c [Å]	107.4, 107.4, 218.6	196.8, 196.8, 65.3	108.2, 108.2, 230.4
Resolution [Å][a]	20.00 - 2.60<br>(2.76 – 2.60)	50.00 - 2.27<br>(2.41 - 2.27)	48.96-3.10<br>(3.29-3.10)
Reflections			
Total	473,592 (75,703)	561,781 (87,917)	663,719 (101,808)
Unique	40,188 (6,358)	66,509 (10,563)	25,633 (4,012)
Multiplicity	12.1 (12.0)	8.4 (8.3)	25.9 (25.4)
Completeness [%]	99.9 (99.7)	99.7 (98.4)	99.9 (99.6)
Mean I/σ(I)	14.33 (0.98)	9.88 (1.11)	16.49 (1.59)
R_{merge}(I) [b)]	0.145 (2.284)	0.205 (1.853)	0.194 (2.125)
R_{meas}(I) [(c)]	0.152 (2.386)	0.218 (1.975)	0.198 (2.169)
$CC_{1/2}$ [%][(d)]	99.9 (58.5)	99.7 (50.9)	99.9 (88.9)
**Refinement**			
Resolution [Å][a]	20.00 - 2.59	50.00 - 2.27	50.00-3.10
Reflections			
Unique	40,006	66,484	25556
Test set [%]	5.0	3.1	5.0
R_{work} [(e)]	0.210 (0.430)	0.197 (0.310)	0.255 (0.372)
R_{free} [(f)]	0.254 (0.470)	0.235 (0.349)	0.305 (0.451)
Contents of A.U.[(g)]			
Non-H atoms	7437	8044	7293
Protein residues/atoms	1008 / 7363	1038/ 7621	997/ 7290
NAD$^+$ molecules	1	4	-
PO/OH molecules	-	2	-
Chloride ion	2	1	-
Water molecules	28	196	3
Mean B factors [Å^2]			
Wilson	71.9	47.9	82.7
Model atoms	81.0	51.8	94.8
Rmsd[(h)] from ideal geometry			
Bond lengths [Å]	0.006	0.007	0.004
Bond angles [°]	0.870	0.949	0.748
Model quality[(i)]			
Overall score	1.87	1.46	1.85
Clash score	8.0	4.1	9.73
Ramachandran favored [%]	97.6	96.9	95.12
Ramachandran outliers [%]	0.1	0.8	0.0
PDB ID	6ZZT	6ZYZ	6ZZ0

[a] Values in parentheses refer to the highest resolution shells.

[b] $R_{merge}(I) = \sum_h \sum_i |I_{ih} - \langle I_h \rangle| / \sum_h \sum_i I_{ih}$, in which $\langle I_h \rangle$ is the mean intensity of symmetry-equivalent reflections h and I_{ih} is the intensity of a particular observation of h.[6]

[c] $R_{meas}(I) = \sum_h [N/(N-1)]^{1/2} \sum_i |I_{ih} - \langle I_h \rangle| / \sum_h \sum_i I_{ih}$, in which $\langle I_h \rangle$ is the mean intensity of symmetry-equivalent reflections h, I_{ih} is the intensity of a particular observation of h and N is the number of redundant observations of reflection h.[6]

[d] $CC_{1/2} = (\langle I^2 \rangle - \langle I \rangle^2) / (\langle I^2 \rangle - \langle I \rangle^2) + \sigma^2_\varepsilon$, in which σ^2_ε is the mean error within a half-dataset.[6]

[e] $R_{work} = \sum_h |F_o - F_c| / \sum F_o$ (working set, no σ cut-off applied).

[f] R_{free} is the same as R_{work}, but calculated on the test set of reflections excluded from refinement.

[g] A.U. – asymmetric unit.
[h] Rmsd – root-mean-square deviation
[i] Calculated with MolProbity.[7]

Table S4. Residues in the active site for SrBDH1 and comparison with the equivalent residues in other members of the family of borneol dehydrogenases.

									Selective?	
	99	146	154	155	159	190	191	211	Borneol	Isoborneol
SrBDH1	G	S	I	A	Y	F	G	M	Yes	Yes
SoBDH1	G	S	I	S	Y	F	G	M	Yes	Yes
SrBDH2	G	S	E	S	Y	C	A	I	Yes	No
SoBDH2	P	S	L	G	Y	Y	A	V	Yes	No
AaADH2	N	S	E	S	Y	G	S	L	No	No
PsBDH	H	S	I	N	Y	Y	M	T	No	No
LiBDH	A	S	G	T	Y	Y	Y	F	No	Yes
AaBDH	E	S	V	A	Y	Y	F	-	No	No
ZsD1	D	S	A	G	Y	Y	A	V	?	?
CoTR	T	S	N	V	Y	G	F	-	?	?
SoBDH3	G	S	L	A	Y	H	G	G	-	-

Table S5. Primers for point mutation

Mutant	Replaced nucleotides	Primers used
G99N	g298a_g299a	5'-gatgctgttcgggttttcaacaatacccgcgttgctgaac-3'
		5'-gttcagcaacgcgggtattgttgaaaacccgaacagcatc-3'
G99H	g298c_g299a	5'-atgctgttcgggtgttcaacaatacccgcgttgctga-3'
		5'-tcagcaacgcgggtattgttgaacacccgaacagcat-3'
G99D	g299a	5'-caaagatgctgttcgggtcttcaacaatacccgcg-3'
		5'-cgcgggtattgttgaagacccgaacagcatctttg-3'
G99T	g298a_g299c	5'-atgctgttcggggtttcaacaatacccgcgttgctga-3'
		5'-tcagcaacgcgggtattgttgaaaccccgaacagcat-3'
F190Y	t572a_c573t	5'-ccggtcagaacaccatacgggctcacgcagtt-3'
		5'-aactgcgtgagcccgtatggtgttctgaccgg-3'
G191M	g574a_g575t_t576g	5'-cgatgccggtcagaaccatgaacgggctcacgcag-3'
		5'-ctgcgtgagcccgttcatggttctgaccggcatcg-3'
G191S	g574a	5'-ccggtcagaacactgaacgggctcacg-3'
		5'-cgtgagcccgttcagtgttctgaccgg-3'
G191F	g574t_g575t	5'-gatgccggtcagaacaaagaacgggctcacgcag-3'
		5'-ctgcgtgagcccgttctttgttctgaccggcatc-3'
M211V	a634g	5'-cccactttgctcacaataccttcaaacatcagcttgctc-3'
		5'-gagcaagctgatgtttgaaggtattgtgagcaaagtggg-3'
M211L	a634t	5'-cccactttgctcaaaataccttcaaacatcagcttgctc-3'
		5'-gagcaagctgatgtttgaaggtattttgagcaaagtggg-3'
V97G	t293g	5'-ctgttcgggccttcaccaatacccgcgttgc-3'
		5'-gcaacgcgggtattggtgaaggcccgaacag-3'
V97C	g292t_t293g	5'-gctgttcgggccttcacaaatacccgcgttgctgaac-3'
		5'-gttcagcaacgcgggtatttgtgaaggcccgaacagc-3'
V97P	g292c_t293c	5'-ctgttcgggccttcaggaatacccgcgttgctgaa-3'
		5'-ttcagcaacgcgggtattcctgaaggcccgaacag-3'
Y159F	a479t	5'-ttgctcgcggtgaagctgtggcccg-3'
		5'-cgggccacagcttcaccgcgagcaa-3'
S146A	a439g_g440c	5'-caatctcggtgcacgcggccgcggtaaaaatgatgc-3'
		5'-gcatcatttttaccgcggccgcgtgcaccgagattg-3'
I196L	a589c	5'-cgtccggcacgaggccggtcagaac-3'
		5'-gttctgaccggcctcgtgccggacg-3'
V97F	g292t	5'-gttcgggccttcaaaaatacccgcgttgctga-3'
		5'-tcagcaacgcgggtatttttgaaggcccgaac-3'
V97Y	g292t_t293a	5'-gctgttcgggccttcataaatacccgcgttgctgaac-3'
		5'-gttcagcaacgcgggtatttatgaaggcccgaacagc-3'
F207W	t623g_t624g	5'-ctttgctcataataccttcccacatcagcttgctcgcctc-3'
		5'-gaggcgagcaagctgatgtgggaaggtattatgagcaaag-3'
C148A	t445g_g446c	5'-cgcaatctcggtggccgcgctcgcggta-3'
		5'-taccgcgagcgcggccaccgagattgcg-3'
G99T+V98C	g292t_t293g_g298a_g299c	5'-gctgttcggggtttcacaaatacccgcgttgctgaaca-3'
		5'-tgttcagcaacgcgggtatttgtgaaaccccgaacagc-3'

3. Nucleotide and amino acid sequences of the enzymes used in this study

a. Amino acid sequences

>AaADH2

MNGVYPHRLLEGKVAIITGGASGFGESTVRLFAKHGAKVVIADIQDQLGLSLCNDLVNKIGDNVIYLHCDVTKES
ENIENTVNTAVSKFGKLDIMFNNAGIPGNLDFTILNSDNENFKRVFDVNVFGSFLGAKHAARVMIPAKRGVILFTS
SVASVLAGESPHSYTVSKHAVIGLMKNLCVELGQYGIRVNCISPGSVSTPLVTTAMGVDKEVVDGILCASAVLK
GVVPTADDVAEAALYLGSDASRYVTGVNLVVDGGYSTTNPTYSRVIKQTFEDLAKKNEGCNGNGVSHAT

>PsBDH

MKLLEGKRIIVTGGAQGIGASVVRAYIAAGATVASMDMNDTLGQQVVSEAGKANPGCKSRYYHCNIADRPEVE
KAFATAAEDMGGLDVMVNVAGVHRHSPPDAIAEELYDMLFRVNVLGTINTNAVAYRLMKGQGIGNIINFGSES
GLTGEINNALYSATKAAVHTWTRNVARQWGPDGIRINAVLPYMVTPMYVDFRNALSSEDLAAHDAATKTDIPL
GGKFGDADKDLAPVMVFLASDASHFMTGQMFPVDGGLIAVR

>SoBDH1

MSSSAVSKRLEGKVAIVTGGASGIGASTVSLFHDHGAKVVIADIQDNLGQTLAGRLGRNISYIHCDVTDENQVRA
LVDATVAKHGGVDIMFSNAGIVEGPTVSIFDADKGALERLLGINLVGGFLAAKHAARVMSPTKKGCIIFTASACT
EVAGISGPGYVASKYGIVGLMKSLAAELGSHGIRANCVSPFGVLTGIAAGDDKTKLMFEGLMSKVGNLKGKILT
ADDVAKAALYLASDEASYVSGVNLVLDGGYSVVNP

>SoBDH2

MATGAANVESPQSLPLRLLGRVALVTGGSSGIGESIVLLFRKHGAKVCIADVQDNQGQRLCETLGGSSDIAFCHC
DVTIEDDVKRAVDFTVDKFGTLDIMVNNAGVSGPPCPDIRDFELSAFDRVFDINVRGVFIGMKHAARIMIPAKKG
SIISISSVASTMGGLGPHAYTGSKHAVLGLTKNVAAELGKHGIRVNCVSPYAVATSLALAHLPEAERTEDTWDDF
RRFVADNANLQGVELTMEDVANAVVFLASDEARYVSGMNLMVDGGFTSTNHALQVFRP

>SrBDH1

MSCNTAVSRRLEGKVAIVTGGASGIGASTVRLFHDHGAKVVIADIQDDLGQTLADRLGRNISYTHCDVTDEDQV
RALVDAAVAKHGGVDIMFSNAGIVEGPNSIFDVDKDELERLMGINLVGAFLAAKHAARVMVPAKKGCIIFTASA
CTEIAGIAGHSYTASKYGIVGLMKSLAVELGSHGIRANCVSPFGVLTGIVPDDEASKLMFEGIMSKVGNLKGKILT
AEDVAVTVLYLASEEASYVSGVNLLVDGGYTVVNPTFINVITAGQS

>SrBDH2

MKMKPESNGLHTSKRLEGKVAIITGGASGFGEATAALFVRHGAKVVIADVQDDRGSALCRDLGLPNQISYVHCD
VTSDADVSAAVDLAVSKYGGLDIMFNNAGIPGGLDFTIVDADNDNFRRVFEVNVYGAFLGAKHAARAMIPARR
GGAILFTASVASAVAGESPHSYAASKHAVVGLMRNLCVELGQHGIRVNAISPCAVATPLLTGTMGVEKAVVEDI
ICASANLKGVVPTAEDVAEAALYLGSDESKFVSGLNLVVDGGYSTTNQSYSRAGTTGSQTLPSASSREPEMG

>SoBDH3

MDSDESKDDMTHHLPSKRLSGKVAVITGGARGIGAATAKAFAENGANVVIADILDEPGAEVAAAIGGKYVHCD
VSVEKDVERAIQVAVDWKGRLDIMFNNAGISGPEGSITNLKMEQLAALLEINLNGVVHGIKHAARAMIEGRNAG
TIICSSSSAATMGGLASHAYTLSKAAILGVARSSACELGLHSIRVNCVSPHGVPSEMLMTAYRRFLGNENLQPQD
VRKIVGEKGSLLRGRGGSMEDVAEAVVFLASDEAGFITGHNLVIDGGYTCASNQMNFIYQE

b. Nucleotide sequence (codon optimized for *E. coli*)

>SoBDH1

ATGAGCAGCAGCGCGGTGAGCAAACGCCTGGAAGGCAAAGTGGCGATTGTGACCGGCGGCGCGAGCGGCA
TTGGCGCGAGCACCGTGAGCCTGTTTCATGATCATGGCGCGAAAGTGGTGATTGCGGATATTCAGGATAAC
CTGGGCCAGACCCTGGCGGGCCGCCTGGGCCGCAACATTAGCTATATTCATTGCGATGTGACCGATGAAAA
CCAGGTGCGCGCGCTGGTGGATGCGACCGTGGCGAAACATGGCGGCGTGGATATTATGTTTAGCAACGCGG
GCATTGTGGAAGGCCCGACCGTGAGCATTTTTGATGCGGATAAAGGCGCGCTGGAACGCCTGCTGGGCATT
AACCTGGTGGGCGGCTTTCTGGCGGCGAAACATGCGGCGCGCGTGATGAGCCCGACCAAAAAAGGCTGCAT
TATTTTTACCGCGAGCGCGTGCACCGAAGTGGCGGGCATTAGCGGCCCGGGCTATGTGGCGAGCAAATATG
GCATTGTGGGCCTGATGAAAAGCCTGGCGGCGGAACTGGGCAGCCATGGCATTCGCGCGAACTGCGTGAGC
CCGTTTGGCGTGCTGACCGGCATTGCGGCGGGCGATGATAAAACCAAACTGATGTTTGAAGGCCTGATGAG
CAAAGTGGGCAACCTGAAAGGCAAAATTCTGACCGCGGATGATGTGGCGAAAGCGGCGCTGTATCTGGCG
AGCGATGAAGCGAGCTATGTGAGCGGCGTGAACCTGGTGCTGGATGGCGGCTATAGCGTGGTGAACCCGTG
A

>SoBDH2

ATGGCTACAGGCGCTGCAAATGTTGAGTCACCACAGTCTCTCCCCTTAAGATTATTAGGGAGAGTTGCTCTG
GTCACCGGAGGTTCGAGTGGCATTGGAGAGAGCATCGTGCTTCTGTTTCGTAAACATGGTGCAAAAGTTTGT
ATAGCCGATGTTCAAGACAACCAAGGGCAACGTCTCTGCGAAACCCTAGGTGGCAGCTCAGACATCGCCTT
TTGCCACTGCGATGTGACAATTGAAGATGATGTCAAGCGTGCAGTGGACTTCACCGTGGACAAGTTCGGTA
CCCTCGACATAATGGTGAACAACGCTGGGGTGTCGGGCCCACCCTGCCCCGATATCCGCGACTTTGAACTCT
CCGCTTTCGACAGGGTCTTCGACATAAACGTGAGAGGGGTTTTCATCGGAATGAAGCACGCGGCTCGCATA
ATGATCCCGGCCAAGAAAGGGTCGATAATATCAATCTCCAGCGTGGCGAGCACTATGGGCGGCTTAGGGCC
TCACGCATACACGGGGTCCAAGCATGCTGTTCTGGGACTCACCAAGAACGTCGCGGCGGAGCTAGGGAAAC
ACGGCATACGCGTGAACTGCGTGTCGCCGTACGCGGTCGCGACTAGCTTGGCGCTGGCGCACTTGCCCGAG
GCGGAGAGGACGGAGGATACGTGGGATGATTTCCGTAGATTTGTGGCGGATAATGCAAACTTGCAGGGAGT
GGAATTGACTATGGAGGATGTGGCGAATGCGGTGGTTTTCTTGGCGAGTGATGAGGCAAGGTATGTAAGCG
GCATGAATCTCATGGTTGATGGAGGCTTCACATCTACAAATCATGCCCTCCAAGTATTTCGTCCCTGA

>SrBDH1

ATGAGCTGCAACACCGCGGTTAGCCGTCGTCTGGAGGGCAAGGTTGCGATTGTTACCGGTGGCGCGAGCGG
TATTGGTGCGAGCACCGTGCGTCTGTTCCACGATCACGGCGCGAAAGTGGTTATCGCGGACATTCAGGACG
ATCTGGGTCAAACCCTGGCGGATCGTCTGGGCCGTAACATCAGCTACACCCACTGCGACGTTACCGACGAG
GATCAGGTTCGTGCGCTGGTTGACGCGGCGGTTGCGAAGCATGGTGGCGTGGACATCATGTTCAGCAACGC
GGGTATTGTTGAAGGCCCGAACAGCATCTTTGACGTGGATAAGGACGAGCTGGAACGTCTGATGGGTATTA
ACCTGGTTGGTGCGTTCCTGGCGGCGAAACATGCGGCGCGTGTGATGGTTCCGGCGAAGAAAGGTTGCATC
ATTTTTACCGCGAGCGCGTGCACCGAGATTGCGGGTATTGCGGGCCACAGCTACACCGCGAGCAAGTATGG
TATCGTTGGCCTGATGAAAAGCCTGGCGGTGGAACTGGGTAGCCACGGCATTCGTGCGAACTGCGTGAGCC
CGTTCGGTGTTCTGACCGGCATCGTGCCGGACGATGAGGCGAGCAAGCTGATGTTTGAAGGTATTATGAGC
AAAGTGGGTAACCTGAAGGGCAAAATCCTGACCGCGGAAGATGTGGCGGTTACCGTGCTGTACCTGGCGAG
CGAGGAAGCGAGCTATGTTAGCGGTGTGAACCTGCTGGTTGACGGTGGCTATACCGTGGTTAACCCGACCT
TTATCAACGTGATTACCGCGGGCCAAAGCTAA

>SrBDH2

ATGAAGATGAAACCGGAGAGCAACGGTCTGCACACCAGCAAGCGTCTGGAAGGCAAAGTTGCGATCATTA
CCGGTGGCGCGAGCGGTTTTGGTGAAGCGACCGCGGCGCTGTTTGTTCGTCACGGTGCGAAGGTGGTTATC
GCGGATGTGCAGGACGATCGTGGTAGCGCGCTGTGCCGTGACCTGGGTCTGCCGAACCAAATTAGCTACGT
GCACTGCGATGTTACCAGCGATGCGGATGTGAGCGCGGCGGTTGATCTGGCTGTGAGCAAATATGGTGGCC
TGGACATCATGTTCAACAACGCGGGCATTCCGGGTGGCCTGGACTTTACCATCGTTGACGCGGATAACGAC
AACTTCCGTCGTGTGTTTGAAGTGAACGTTTACGGTGCGTTCCTGGGCGCGAAACATGCGGCGCGTGCGATG
ATCCCGGCGCGTCGTGGTGGCGCGATTCTGTTTACCGCGAGCGTTGCGAGCGCGGTTGCGGGCGAGAGCCC

GCACAGCTATGCGGCGAGCAAACACGCGGTGGTTGGCCTGATGCGTAACCTGTGCGTTGAACTGGGTCAGC
ACGGCATCCGTGTTAACGCGATTAGCCCGTGCGCGGTTGCGACCCCGCTGCTGACCGGTACCATGGGCGTG
GAGAAGGCGGTGGTTGAAGATATCATTTGCGCGAGCGCGAACCTGAAAGGTGTGGTTCCGACCGCGGAGG
ATGTTGCGGAAGCGGCGCTGTACCTGGGTAGCGACGAGAGCAAGTTCGTGAGCGGCCTGAACCTGGTGGTT
GACGGTGGCTACAGCACCACCAACCAGAGCTATAGCCGTGCGGGTACCACCGGCAGCCAGACCCTGCCGAG
CGCGAGCAGCCGTGAGCCGGAAATGGGTTAA

>SoBDH3

ATGGACAGCGATGAAAGCAAGGACGATATGACCCACCACCTGCCGAGCAAGCGTCTGAGCGGCAAAGTGG
CGGTTATCACCGGTGGCGCGCGTGGTATTGGTGCGGCGACCGCGAAAGCGTTTGCGGAGAACGGCGCGAAC
GTGGTTATCGCGGACATTCTGGATGAGCCGGGTGCGGAAGTGGCGGCGGCGATTGGTGGCAAGTACGTTCA
CTGCGACGTGAGCGTTGAGAAAGATGTGGAACGTGCGATCCAGGTGGCGGTTGACTGGAAGGGTCGTCTGG
ATATCATGTTTAACAACGCGGGTATTAGCGGCCCGGAAGGTAGCATCACCAACCTGAAGATGGAGCAACTG
GCGGCGCTGCTGGAAATCAACCTGAACGGCGTGGTTCACGGTATTAAGCATGCGGCGCGTGCGATGATTGA
GGGTCGTAACGCGGGTACCATCATTTGCAGCAGCAGCAGCGCGGCGACCATGGGTGGCCTGGCGAGCCATG
CGTACACCCTGAGCAAAGCGGCGATTCTGGGCGTGGCGCGTAGCAGCGCGTGCGAACTGGGTCTGCACAGC
ATCCGTGTGAACTGCGTTAGCCCGCACGGCGTTCCGAGCGAGATGCTGATGACCGCGTATCGTCGTTTCCTG
GGTAACGAAAACCTGCAGCCGCAAGACGTGCGTAAGATTGTTGGCGAGAAAGGTAGCCTGCTGCGTGGTCG
TGGTGGCAGCATGGAGGACGTGGCGGAAGCGGTGGTTTTCCTGGCGAGCGATGAAGCGGGCTTTATTACCG
GTCACAACCTGGTTATCGATGGTGGCTACACCTGCGCGAGCAACCAGATGAACTTTATCTATCAAGAGTAA

4. Complete name and accession numbers of SDR used in phylogenetic tree

LiBDH: borneol dehydrogenase from *Lavandula intermedia* (AFV30207.1[a])

AaBDH: borneol dehydrogenase from *Artemisia annua* (ANJ65052.1[a])

AaADH2: alcohol dehydrogenase from *Artemisia annua* (ADK56099.1[a])

PsBDH: borneol dehydrogenase from *Pseudomonas sp. TCU-HL1* (WP_032492645.1[a])

SoBDH1: borneol dehydrogenase 1 from *Salvia officinalis L.* (MT525100[a])

SoBDH2: borneol dehydrogenase 2 from *Salvia officinalis L.* (MT525099 [a])

SoBDH3: alcohol dehydrogenase 3 from *Salvia officinalis L.*

SrBDH1: alcohol dehydrogenase 1 from *Rosmarinus officinalis* (MT857224[a])

SrBDH2: alcohol dehydrogenase 2 from *Rosmarinus officinalis*

PgADH: short-chain alcohol dehydrogenase from *Panax ginseng* (ACL37155.1[a])

PaADH: alcohol dehydrogenase from *Prunus armeniaca* (ABZ79222.1[a])

CmADH: putative alcohol dehydrogenases from *Cucumis melo* (ABC02082.1[a])

AtADH: putative dehydrogenase from *Arabidopsis thaliana* (AAM65725.1[a])

DsTR: tropinone reductase-II from *Datura stramonium* (AAA33282.1[a])

CoTR: Tropinone reductase from *Cochlearia officinalis*

At2g29330: Tropinone reductase from *Arabidopsis thaliana* (Q9ZW16[b])

At2g29150: Tropinone reductase from *Arabidopsis thaliana* (Q9ZW03[b])

At2g29350: Senescence-associated protein from *Arabidopsis thaliana* (Q9ZW18[b])

MMR: Menthol dehydrogenase from *Mentha x piperita* (AAQ55960.1[a])

MNR: neomenthol dehydrogenase from *Mentha x piperita* (AAQ55959.1[a])

SAD-C: short-chain alcohol dehydrogenase from *Pisum sativum* (AAF04253.1[a])

DlHSDH: 3-beta-hydroxysteroiddehydrogenase from *Digitalis lanata* (Q93Y47[b])

PeSDH: Secoisolariciresinol dehydrogenase from *Podophyllum peltatum* (AAK38664.1[a])

FiSDH: secoisolariciresinol dehydrogenase from *Forsythia x intermedia* (AAK38665.1[a])

HsCR: carbonyl reductase from *Homo sapiens* (AAA52070.1[a])

SsHSDH: 20-beta-hydroxysteroid dehydrogenase from *Sus scrofa* (AAA30980.1[a])

CgCR: Carbonyl reductase from *Cricetulus griseus* (BAB07797.1[a])

MpIDH: (-)-isopiperitenol dehydrogenase *Mentha x piperita* (AAU20370.1[a])

CrADH1: Alcohol dehydrogenase 1 from *Catharanthus roseus* (5O98_1[c])

salR: Salutaridine reductase from *Papaver somniferum* (3O26_1[c])

ZSD1: Short-chain dehydrogenase/reductase 1 from *Zingiber zerumbet* (BAK09296.1[a])

ZmSDP: Sex determination protein tasselseed-2 from *Zea mays* (ACG37730.1[a])

CsADH: Short chain alcohol dehydrogenase from *Citrus sinensis* (ADH82118.1[a])

StDR: Short-chain dehydrogenase/reductase from *Solanum tuberosum* (AAT75153.1[a])

CbHCDH: (S)-6 beta-hydroxycineole dehydrogenase from *Citrobacter braakii* (ACX31575.1[a])

RsGDH: Galactitol dehydrogenase from *Rhodobacter sphaeroides* (ACM89305.1[a])

AaFDH: Farnesol dehydrogenase 1 from *Aedes aegypti* (ADB03639.1[a])

AaDH: Broad substrate reductase/dehydrogenase *Artemisia annua* (ACZ34296.1[a]).

[a]NCBI, [b]Uniprot, [c]PDB

5. Commands used for couple moves protocol

~/Rosetta/main/source/bin/coupled_moves.linuxgccrelease –s pdb_file –resfile res_file –database ~/Rosetta/main/database –mute protocols.backrub.BackrubMover –extra_res_fa params_file –ex1 –ex2 –extrachi_cutoff 0 –nstruct 99 –coupled_moves::mc_kt 0.6 –coupled_moves::ntrials 1000 –coupled_moves::initial_repack false –coupled_moves::ligand_mode true –coupled_moves::ligand_weight N

References

[1] D. Liebschner, P. V. Afonine, N. W. Moriarty, B. K. Poon, O. V. Sobolev, T. C. Terwilliger, P. D. Adams, *Acta Crystallogr. Sect. D Struct. Biol.* **2017**, *73*, 148–157.

[2] N. Ollikainen, R. M. de Jong, T. Kortemme, *PLoS Comput. Biol.* **2015**, *11*, 1–22.

[3] D. R. Polichuk, Y. Zhang, D. W. Reed, J. F. Schmidt, P. S. Covello, *Phytochemistry* **2010**, *71*, 1264–1269.

[4] I. Drienovská, D. Kolanovi, A. Chánique, V. Sieber, M. Hofer, R. Kourist, *Phytochemistry* **2020**, *172*, DOI 10.1016/j.phytochem.2019.112227.

[5] H. Tsang, J.-L. Huang, Y.-H. Lin, K. Huang, P. Lu, G. Lin, A. A. Khine, A. Hu, H.-P. Chen, *Appl. Environ. Microbiol.* **2016**, *82*, 6378–6385.

[6] P. A. Karplus, K. Diederichs, *Science (80-.).* **2012**, *336*, 1030–1033.

[7] V. B. Chen, J. R. Wedell, R. K. Wenger, E. L. Ulrich, J. L. Markley, *J. Biomol. NMR* **2015**, *63*, 77–83.
